# Novelty as a drive of human exploration in complex stochastic environments

**DOI:** 10.1073/pnas.2502193122

**Published:** 2025-09-25

**Authors:** Alireza Modirshanechi, Wei-Hsiang Lin, He A. Xu, Michael H. Herzog, Wulfram Gerstner

**Affiliations:** ^a^School of Life Sciences, Brain-Mind Institute, École Polytechnique Fédérale de Lausanne (EPFL), Lausanne 1015, Switzerland; ^b^School of Computer and Communication Sciences, École Polytechnique Fédérale de Lausanne (EPFL), Lausanne 1015, Switzerland; ^c^Helmholtz Munich, Neuherberg 85764, Germany; ^d^Max Planck Institute for Biological Cybernetics, Tübingen 72012, Germany

**Keywords:** exploration, human behavior, reinforcement learning, information-seeking

## Abstract

In order to find extrinsic rewards, humans explore their environment even if exploration requires several intermediate, reward-free decisions. It has been hypothesized that intrinsic rewards, such as novelty, surprise, or information gain, guide this reward-free exploration. However, in artificial agents, different intrinsic reward signals induce exploration strategies that respond differently to stochasticity. In particular, some strategies are vulnerable to the “noisy TV problem,” i.e., an attraction to irrelevant stochastic stimuli. Here, we ask whether humans exhibit a similar attraction to reward-free stochasticity. We design a multistep decision-making paradigm in which participants search for rewarding states in a complex environment containing a highly stochastic but reward-free subregion. We show that i) participants persistently explore the stochastic subregion, and ii) their decisions are best explained by a novelty-driven exploration strategy, compared to alternatives driven by information gain or surprise. Our findings suggest that novelty and extrinsic rewards jointly control human exploration in complex environments.

## Significance

Would you choose to complete a task in a few seconds for a guaranteed reward, or spend half an hour exploring unknown paths that may or may not lead to something better? Using a multistep decision-making task and computational modeling, we show that when searching for monetary rewards, humans tend to overexplore, being drawn aside to novel parts of the environment even when exploration is unhelpful. Our model explains this behavioral pattern of humans with high precision and suggests that optimism about finding greater rewards governs the interplay between novelty and extrinsic incentives. These findings may help explain real-world behaviors, as diverse as social media overuse or analysis paralysis, where people continue to explore despite diminishing returns or increasing costs.

Humans frequently search for more valuable rewards (e.g., more nutritious foods or better-paid jobs) than those currently available (1–3). However, the computational and algorithmic nature of this exploratory behavior has remained highly debated (4–6). State-of-the-art models of human exploration use intrinsically motivated reinforcement learning (RL) algorithms (7–10) that, initially inspired by research in psychology (11, 12), have been designed to solve complex machine learning tasks with sparse “extrinsic” rewards (13–19). These algorithms use internally generated signals like “novelty,” “surprise,” or “information gain” as “intrinsic” rewards to guide exploratory action choices (11). However, different intrinsic rewards result in different exploration strategies (20, 21). An unresolved yet crucial puzzle in neuroscience and psychology is identifying the type of intrinsic reward that drives exploration in humans (9, 10).

Resolving this puzzle primarily requires advances in experimental design. Experimental studies of human exploration have been mainly limited to simple experimental paradigms where a single action (or at most a pair of actions) is sufficient for reaching an extrinsic reward (22–28) or information (29–33). These tasks are principally different from exploration in the real world, where reaching a “goal” requires several intermediate actions with no explicit progress feedback (9). This has recently led to major concerns about the reliability and relevance of these tasks for characterizing human exploratory behavior (34–36). Studying exploration in multistep tasks (37, 38) is hence pivotal for understanding and modeling human exploration (9, 39, 40).

Compared to traditional experimental paradigms with homogeneously distributed stochasticity (41, 42), multistep environments with a localized stochastic component have an important advantage: they enable the dissociation of exploration strategies based on different intrinsic rewards. Specifically, machine learning research has shown that intrinsically motivated RL agents are prone to distraction by stochasticity, i.e., they are attracted to novel, surprising, or just noisy states independently of whether or not these states are rewarding (43) [the so-called “noisy TV” problem (20, 21)]. However, the extent of this distraction varies between algorithms and depends on the type of intrinsic reward (44–48). Artificial RL agents seeking information gain eventually lose their interest in stochasticity when exploration yields no further information (20, 21); in contrast, RL agents seeking surprise or novelty exhibit a persistent attraction by stochasticity (20, 21).

Here, we ask i) whether humans are distracted in the same situations as intrinsically motivated RL agents and, if so, ii) whether this distraction vanishes (similar to seeking information gain) or persists (similar to seeking surprise or novelty) over time.

## Results

**Fig. 1.**
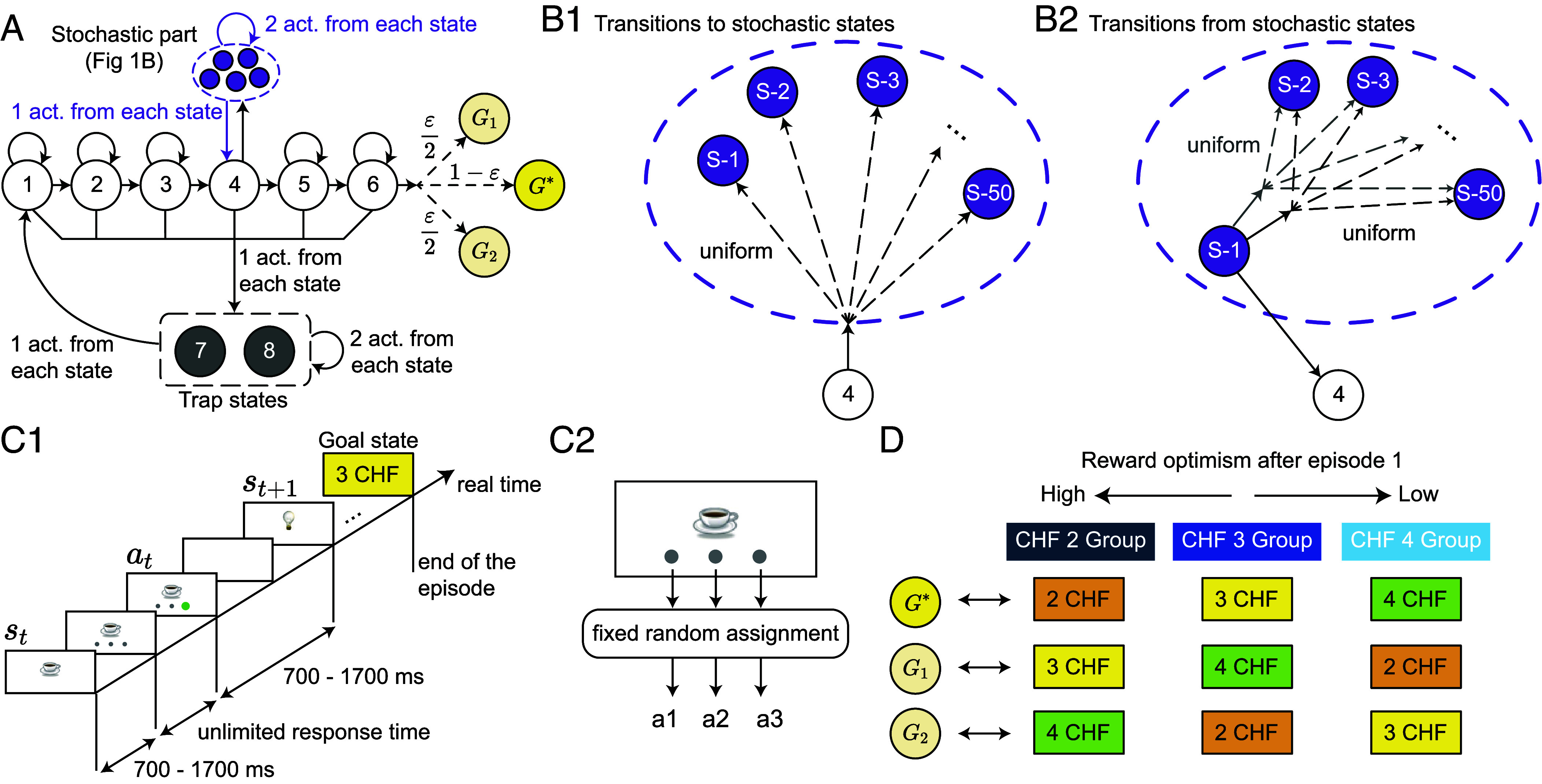
Experimental paradigm. (*A*) Structure of the environment; only 5 out of the 50 stochastic states are shown (dashed oval; see *B*). Each circle represents a state and each solid arrow an action. All actions except those to the stochastic part or to the goal states are deterministic. Dashed arrows indicate random transitions; values (e.g., 1−ε) show the probabilities of each transition. We chose ε≪1 (*Materials and Methods*). (*B*) Zoom on stochastic transitions between states S-1 to S-50 inside the dashed oval. (*B*1) In state 4, one action takes agents randomly (with uniform distribution) to one of the stochastic states. (*B*2) In each stochastic state (e.g., state S-1 in the figure), one action (always the same) takes agents back to state 4 and two actions to another randomly chosen stochastic state. (*C*) Timeline of one episode in human experiments (*C*1). The states were represented by images on a computer screen and actions by disks below each image. The assignment of images to states and disks to actions was random but fixed throughout the experiment (*C*2). An episode ended when a goal image (i.e., “3 CHF” image in this example) was found. (*D*) Human participants were informed that there were three goal states in the environment and that these goal states had different monetary values of 2 Swiss Franc (CHF), 3 CHF, and 4 CHF. For each participant, these monetary reward values were randomly assigned to different goal locations (i.e., $G^{*}$, $G_{1}$, and $G_{2}$ in *A*) at the beginning of the experiment (without informing them); the assignment was fixed throughout the experiment. Hence, $G^{*}$ had a different value for different participants, resulting in three groups of participants with different levels of reward optimism during episodes 2 to 5 (i.e., after finding $G^{*}$ for the first time). See *Materials and Methods*.

We designed an experimental paradigm in which human participants explored an environment comprising 61 states, including three goal states (Fig. 1 *A* and *B*). Three actions were available in each of the 58 nongoal states, and agents could move from one state to another by choosing these actions (arrows in Fig. 1 *A* and *B*). We use the term “agents” to refer to either human participants or agents simulated by RL algorithms. In the human experiments, states were represented by images on a computer screen and actions by three disks below each image (Fig. 1*C*); for RL agents, both states and actions were abstract entities, i.e., we considered RL in a tabular setting (49). The assignment of images to states and disks to actions was random but fixed throughout the experiment (Fig. 1*C*2). Agents were informed that there were three different goal states in the environment ($G^{*}$, $G_{1}$, or $G_{2}$ in Fig. 1*A*) and that their task was to find a goal state 5 times; see *SI Appendix* for how this information was incorporated in the RL algorithms. Neither human participants nor RL agents were aware of the total number of states or the structure of the environment (i.e., how states were connected).

The 58 states of the environment were classified into three groups: progressing states (1 to 6 in Fig. 1*A*), trap states (7 and 8 in Fig. 1*A*), and stochastic states (S-1 to S-50 in Fig. 1*B*, shown as a dashed oval in Fig. 1*A*). In each progressing state, one action (“progressing” action) brought agents one step closer to the goals, while another (“bad” action) brought them to one of the trap states. The third action in states 1 to 3 and 5 to 6 was a “self-looping” action that made agents stay in the same state. Except for the progressing action in state 6, all these actions were deterministic, meaning that they always led to the same next state. The progressing action in state 6 was almost deterministic: it took participants to the “likely” goal state $G^{*}$ with a probability of 1−ε and to the “unlikely” goal states $G_{1}$ and $G_{2}$ with equal probabilities of $\frac{\varepsilon }{2}\ll 1$. In state 4, instead of a self-looping action, there was a “stochastic” action that took agents to a randomly chosen (with equal probability) stochastic state (Fig. 1*B*1). In each stochastic state, one fixed action (e.g., the left disk) reliably took agents back to state 4, and two stochastic actions took them to another randomly chosen stochastic state (Fig. 1*B*2). In each trap state, all three actions were deterministic: two actions brought agents to either the same or the other trap state and one action to state 1.

The stochastic part of the environment—which mimics the main features of a noisy TV (43)—is the crucial difference to existing paradigms (37, 38, 50, 51). Without the stochastic part, all types of intrinsic reward would help agents avoid the trap states and find the goal (37). Hence, intrinsic rewards would help exploration before and not harm exploitation after finding a goal. However, the stochastic part dissociates exploratory behaviors driven by different intrinsic rewards; we elaborate on these differences in later sections (see ref. 20 and *SI Appendix*).

### Reward Optimism as an Incentive to Explore.

We recruited 63 human participants and instructed them to perform our task for five episodes: each episode began by initializing participants at state 1 or 2 and ended when they reached any one of the three goal states (i.e., $G^{*}$, $G_{1}$, and $G_{2}$). We chose a small enough ε (Fig. 1*A*) to safely assume that all participants would visit only $G^{*}$ while being aware that $G_{1}$ and $G_{2}$ existed.

To further motivate exploration, we informed human participants that there were three different possible reward states corresponding to values of 2 Swiss Franc (CHF), 3 CHF, and 4 CHF, represented by three different images (see *Materials and Methods* for details and *SI Appendix* for incorporating this information in the RL algorithms). At the beginning of the experiment, we randomly assigned the three different reward values to the goal states $G^{*}$, $G_{1}$, and $G_{2}$, separately for each participant (without informing them), and kept the assignment fixed throughout the experiment (Fig. 1*D*). Following this random assignment, and after excluding 6 participants from further analyses (see *Materials and Methods* for criteria), $G^{*}$ held different reward values across participants: 21 of 57 participants were assigned to environments with 2 CHF reward value for $G^{*}$, 19 participants to environments with 3 CHF reward value for $G^{*}$, and 17 participants to environments with 4 CHF reward value for $G^{*}$. In the following, we refer to each group by their reward value of $G^{*}$, e.g., the 3 CHF group is the group of human participants who had a reward value of 3 CHF for $G^{*}$ (Fig. 1*D*).

The resulting three groups of human participants were characterized by three different levels of “reward optimism” in episodes 2 to 5, where we define reward optimism as the expectancy of finding a goal of higher value than the one already discovered (Fig. 1*D*); we note that reward optimism in our experiment is closely linked to but independent of general optimism in psychology (52). Hence, even though all participants had received the same instructions, the 4 CHF group did not have any monetary incentive to explore further in episodes 2 to 5, whereas the 2 CHF group had a high monetary incentive to explore and find a higher reward in episodes 2 to 5. Therefore, we expected participants in the 2 CHF group to continue searching for more valuable goals in episodes 2 to 5. In the following sections, we characterize this search behavior, with the aim of identifying its dominant drive.

### Human Participants Persistently Explore the Stochastic Part.

**Fig. 2.**
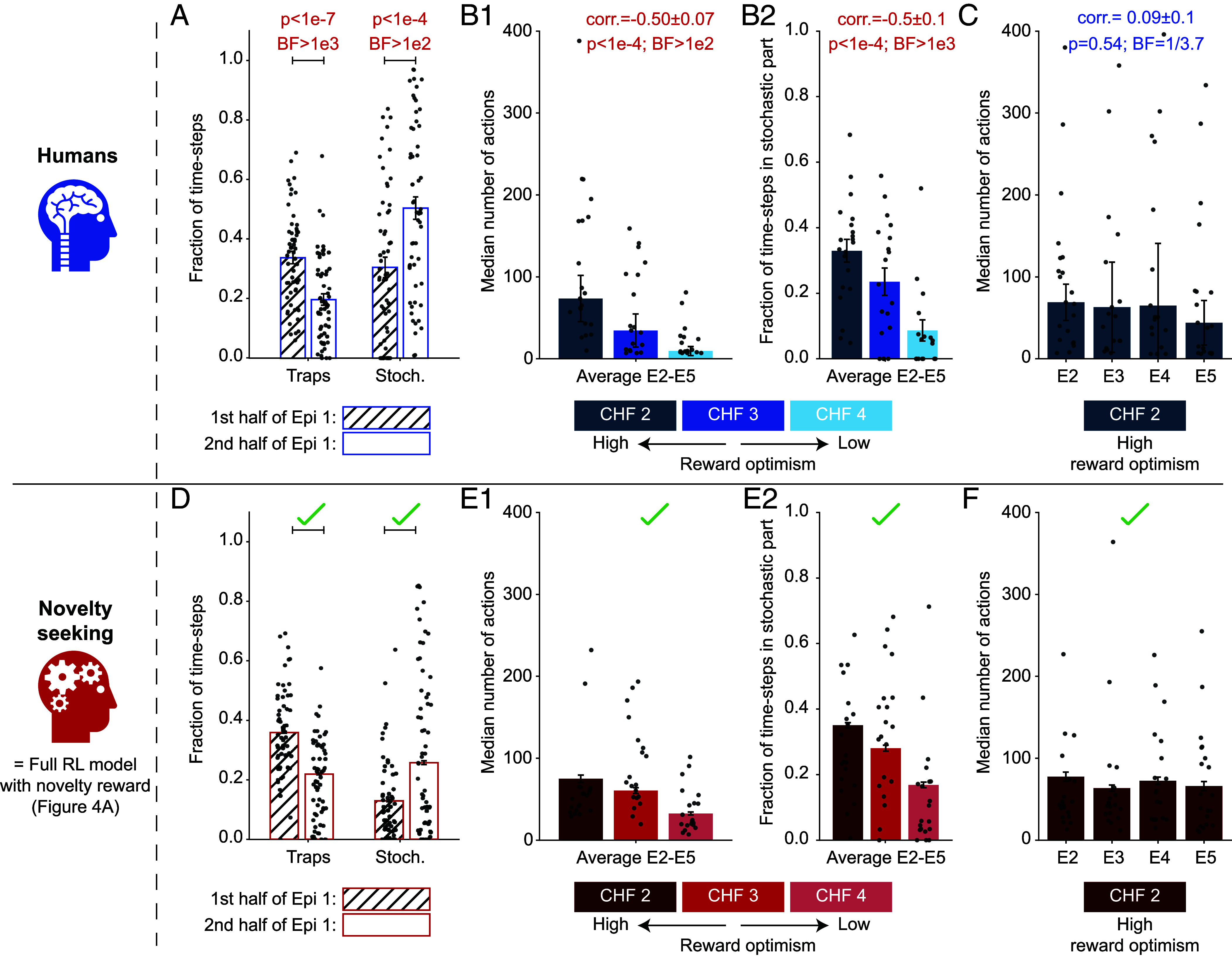
Human participants persistently explore the stochastic part. (*A*) Participants spent less time in the trap states (one-sample *t* test; t=−6.35; 95%CI =(−0.186,−0.097); DF =56) and more time in the stochastic part (t=4.25; 95%CI =(0.073,0.203); DF =56) during the 2nd half of episode 1 (E1) than during the 1st half. Error bars show the SEM. (*B*) Search duration in episodes 2 to 5. (*B*1) Median number of actions over episodes 2 to 5 for the three different groups: 2 CHF (dark), 3 CHF (medium), and 4 CHF (light). Error bars show the SE of the median (SEMed; evaluated by bootstrapping). The Pearson correlation between the search duration and the goal value is negative (correlation test; t=−4.2; 95%CI =(−0.67,−0.27); degree of freedom (DF) =55; *Materials and Methods*). (*B*2) Average fraction of time-steps spent in the stochastic part of the environment during episodes 2 to 5. The Pearson correlation between the fraction of time-steps spent in the stochastic part and the goal value is negative (correlation test; t=−4.7; 95%CI =(−0.70,−0.32); DF =55; *Materials and Methods*). Error bars show the SEM. (*C*) Median number of actions in episodes 2 to 5 for the 2 CHF group. A Bayes Factor (BF) of 1/3.7 in favor of the null hypothesis (53) suggests a zero Pearson correlation between the search duration and the episode number (one-sample *t* test on individual correlations; t=0.63; 95%CI =(−0.20,0.37); DF =20). Error bars show the SEMed. (*D*–*F*) Posterior predictive check (PPC): simulating novelty-seeking RL in our experimental paradigm replicates the main qualitative patterns of the participants’ behavior (see Fig. 5 for quantification across 43 summary statistics). Panels *D*–*F* correspond to panels *A*–*C*, respectively, and illustrate the same summary statistics but for 1,500 simulated novelty-seeking agents. We note that these results must be seen only as post hoc confirmation of the fitted novelty-seeking algorithm—rather than a priori prediction. Single dots in all panels show the data of individual human participants (*A*–*C*) or a subset (20 per group) of simulated participants (*D*–*F*). Red *P*-values in *A*–*C*: significant effects with False Discovery Rate controlled at 0.05 (54) (*Materials and Methods*). Red BFs in *A*–*C*: significant evidence in favor of the alternative hypothesis (BF ≥ 3). Blue BFs in *A*–*C*: significant evidence in favor of the null hypothesis (BF ≤ 1/3).

We first studied the behavior of human participants without explicit computational modeling. During the 1st episode, all three groups of participants (i.e., 2 CHF, 3 CHF, and 4 CHF) had to explore the environment until they found the goal state $G^{*}$ for the first time. Throughout this exploration, they received no intermediate reward or progress feedback. Nevertheless, the participants learned to avoid the trap states (Fig. 2*A*, *Left*) and were attracted to exploring the stochastic part of the environment (Fig. 2*A*, *Right*). This suggests that participants used a guided exploration strategy (as opposed to a random exploration strategy; see *SI Appendix*).

After finding the goal $G^{*}$ for the 1st time (i.e., at the beginning of episode 2), each participant had effectively two options: i) attempt to return to the discovered goal state $G^{*}$ (exploitation) or ii) search for the other goal states $G_{1}$ and $G_{2}$ (exploration). We quantified the extent of the exploratory behavior during episodes 2 to 5 by the search duration (i.e., the number of actions taken before returning to the discovered goal state; Fig. 2*B*1) and the fraction of time-steps spent in the stochastic part (Fig. 2*B*2). Both of these quantities were negatively correlated with the reward value of $G^{*}$, e.g., the 2 CHF group had a longer search duration and spent more time in the stochastic part than the other two groups. Nevertheless, we still found a nonnegligible exploration of the stochastic part by some participants in the 4 CHF group (Fig. 2*B*2, light blue), even though they had already found the goal state with the highest reward value. These observations i) support the hypothesis that a higher degree of reward optimism leads to higher exploration in human participants and ii) imply that human exploratory behavior is guided toward the stochastic part of the environment, even when there is no monetary incentive for exploration (see next section).

The behavior of the 2 CHF group is particularly interesting, as they were, by design, the most optimistic group about finding higher rewards. The 2 CHF group exhibited a constant search duration over episodes 2 to 5 (zero correlation between the search duration and episode index confirmed by Bayesian hypothesis testing (53); Fig. 2*C*). This implies that they persistently explored the stochastic part, even though it would have been theoretically possible to infer the structure of the environment and decrease exploration over time—as shown by “optimal” agents seeking information gain (see ref. 20 for a review and *SI Appendix* for simulations). Collectively, these results indicate that human exploration is neither random nor theoretically optimal (*Discussion*).

### Human Participants Successfully Learned the Environment’s Structure.

**Fig. 3.**
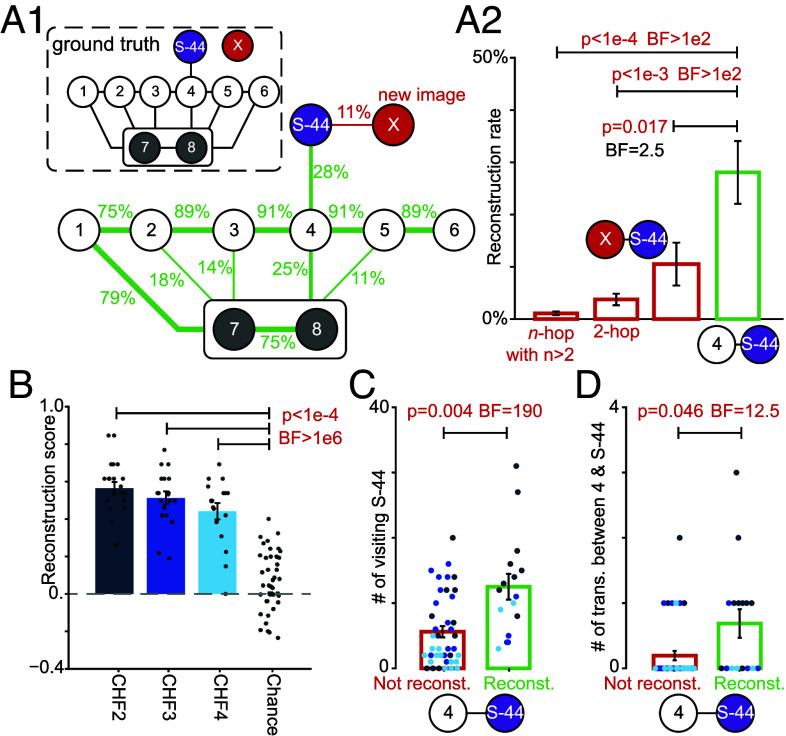
Human participants successfully reconstructed the environment’s structure. At the end of the experiment, participants were shown images of progressing states (1 to 6), trap states (7 to 8), one stochastic state (S-44), and a new image (X). All images were presented simultaneously in a pseudorandom layout, and participants were asked to draw transitions between them (*Materials and Methods*). (*A*) Average reconstruction across participants. (*A*1) Link labels show the proportion of participants who drew each connection (only links >10% shown). *Inset*: Ground truth. (*A*2) The reconstruction rate for the link between state 4 and S-44 was significantly higher than for erroneous links between S-44 and X (one-sample *t* test; t=2.46; 95%CI =(0.03,0.32); DF =56) as well as the 2-hop (t=3.88; 95%CI =(0.12,0.37); DF =56) and n-hop connections (n>2; t=4.44; 95%CI =(0.15,0.39); DF =56). (*B*) Reconstruction scores (range: −1 to +1) were significantly above chance in all reward groups: 2 CHF (one-sample *t* test against 0; t=16.9; 95%CI =(0.49,0.63); DF =20), 3 CHF (t=15.1; 95%CI =(0.44,0.58); DF =18), and 4 CHF groups (t=10.0; 95%CI =(0.35,0.53); DF =16). (*C* and *D*) Participants who reconstructed the link between state 4 and the stochastic state S-44 had visited S-44 significantly more often than those who did not (*C*; unequal variances *t* test; t=3.20; 95%CI =(2.4,11.4); DF =20.9); they had also experienced the transitions between states 4 and S-44 significantly more often than those who did not (*D*; unequal variances *t* test; t=2.14; 95%CI =(0.01,0.97); DF =18.3). Red *P*-values in *B*–*D*: significant effects with false discovery rate controlled at 0.05 (54) (*Materials and Methods*). Red BFs in *B*–*D*: significant evidence in favor of the alternative hypothesis (BF ≥ 3). Error bars in *B* and *C*: SEM. Single dots in *B*–*D*: data of individual participants (color-coded based on their reward group in *C* and *D*); for random drawing in *B* (Chance), we showed only 40 out of 1,000 samples.

Thus far, we have shown that human participants exhibited a persistent attraction to the stochastic part in episodes 2 to 5, a behavioral pattern that is theoretically suboptimal. However, an implicit premise of our conclusion is that participants had learned the environment’s structure well enough to know how to return to $G^{*}$ in episodes 2 to 5. To test this premise, we next analyzed whether participants could reconstruct the environment’s structure at the end of the experiment (Fig. 3). After finishing the experiment, participants were asked to reconstruct a map of the environment by connecting the images of different states (Fig. 3*A*; *Materials and Methods*). All three groups of participants achieved an above-chance reconstruction score (Fig. 3*B*; *Materials and Methods*). Each individual link along the path from the trap states to state 6 was correctly reconstructed by at least 75% of participants (Fig. 3*A*1), and 30 out of 57 participants successfully reconstructed the entire path. This implies that, by the end of the experiment, participants had built an explicit mental path for reaching the goal state $G^{*}$.

The images presented to participants also included one of the stochastic states (S-44) and a new image (X) that did not belong to the 58 states of the environment. Almost one-third of the participants successfully reconstructed the connection between state 4 and S-44 (Fig. 3*A*1), while none linked state 4 and the new image X. The 28% reconstruction rate for the link between state 4 and S-44 was significantly higher than both the rate of erroneously connecting the novel image to S-44 and the baseline error rates for other nonexisting connections, where links drawn by the participant corresponded to a 2-hop or n-hop path (with n>2; Fig. 3*A*2). These results suggest that the 28% reconstruction rate reflects genuine learning of the state transition, rather than participants’ general, baseline tendency to connect different images.

Importantly, while reconstructing the link between states 4 and S-44 indicates that the participant had learned the transition from state 4 to some stochastic states, not reconstructing this link can be due to reasons other than a lack of understanding of the environment’s structure. For example, some participants might have ignored this link because they thought it was unimportant as it was not on the path to rewards, because they could not remember this very specific stochastic state, or because they never experienced a transition between state 4 and S-44. In fact, we observed that participants who reconstructed the link between states 4 and S-44 had visited state S-44 more frequently than those who did not (Fig. 3*C*). Strikingly, half of the participants who reconstructed the link had never directly experienced this specific transition (Fig. 3*D*). This indicates that these participants had learned the structure so thoroughly that they could generalize and reconstruct a link they had never directly encountered.

Overall, these results provide direct evidence that human participants were able to reconstruct a step-by-step map of the environment—despite the unprecedented complexity of the environment compared to other behavioral RL paradigms (42, 50). Hence, these results complement recent findings on human graph learning (55–57) and, most importantly, imply that participants’ theoretically suboptimal exploration strategy is not an obvious consequence of poor graph learning.

### Computational Modeling of Human Exploration.

**Fig. 4.**
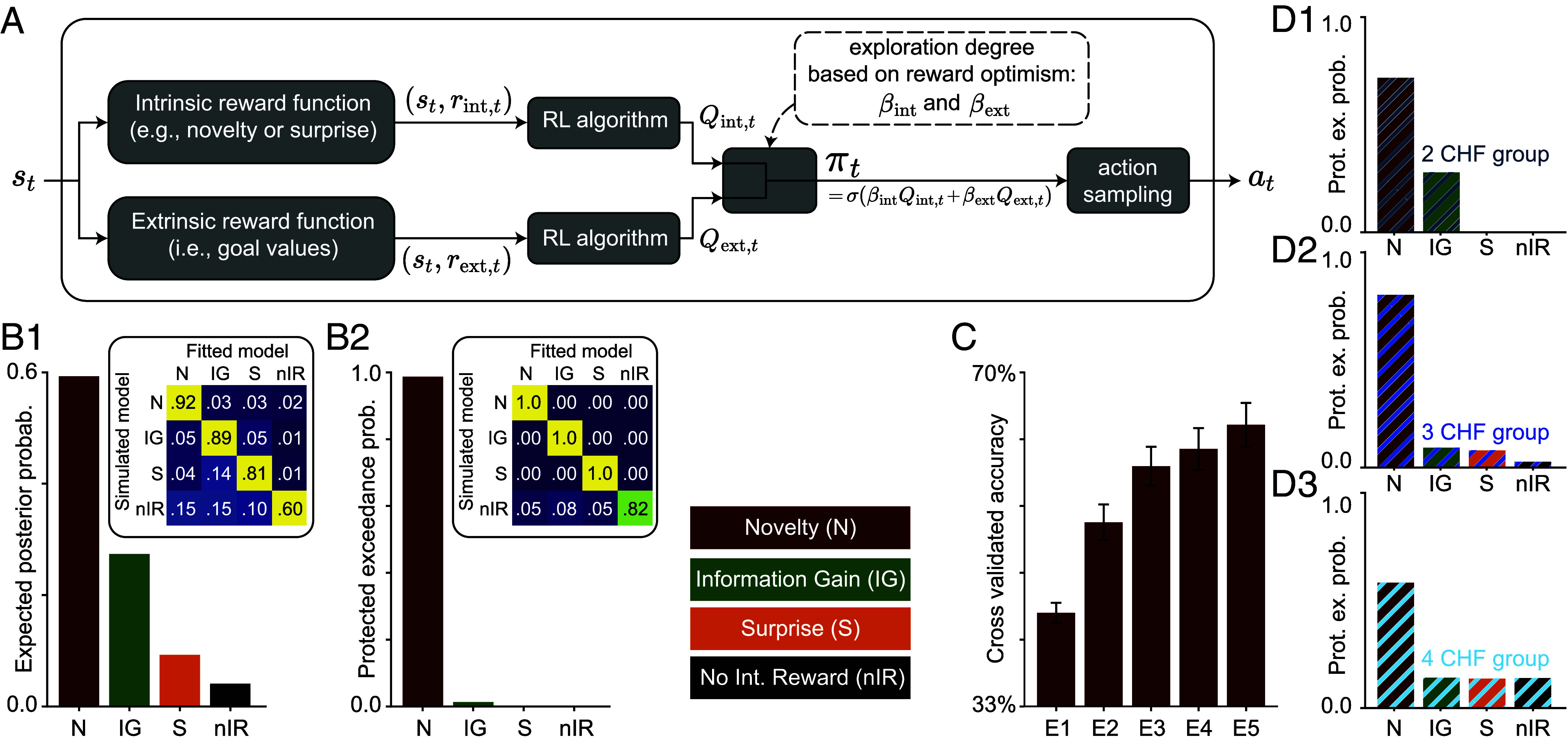
Novelty-seeking is the most accurate model of human behavior. (*A*) Block diagram of the intrinsically motivated RL algorithm for modeling human behavior. Given the state $s_{t}$ at time t, the intrinsic reward $r_{\text{int},t}$ (e.g., novelty) and the extrinsic reward $r_{\text{ext},t}$ (i.e., the monetary reward value of $s_{t}$) are evaluated and passed to two identical (except for the reward signals) parallel RL algorithms. The two algorithms compute two sets of Q-values, one for seeking intrinsic reward $Q_{\text{int},t}$ and one for seeking extrinsic reward $Q_{\text{ext},t}$. The weighted sum of the Q-values is used in a softmax function σ as the action-selection policy $\pi_{t}$. The next action $a_{t}$ is selected by sampling from $\pi_{t}$. See *SI Appendix* for details. (*B*) Bayesian model comparison: human participants’ action choices are best explained by novelty-seeking (N) compared to seeking information gain (IG), seeking surprise (S), or exploration with no intrinsic reward (nIR). (*B*1) The expected posterior probability quantifies the proportion of participants whose behavior is best explained by each algorithm (65) (regarding cross-validated log-likelihoods; *SI Appendix*). (*B*2) Protected exceedance probability (66) quantifies the probability of each model being more frequent than the others among participants. *Insets* show confusion matrices from the model recovery (67) (*SI Appendix*); we could always recover the model that had generated the data, using almost the same number of simulated participants (60) as human participants (57). (*C*) Cross-validated accuracy rate of novelty-seeking in predicting individual actions of human participants. The chance level is 33%. Error bars show the SEM. Novelty-seeking allows above-chance prediction of each participant’s actions. (*D*) Protected Exceedance Probability (as in *B*2) for participants in the 2 CHF (*E*1), 3 CHF (*E*3), and 4 CHF (*E*4) groups. Novelty-seeking is the most frequent model of behavior across and within groups.

To gain insights into the algorithmic form of human exploration, we modeled human participants as intrinsically motivated RL agents that move in an environment with an unknown number of states, seeking both extrinsic and intrinsic rewards (Fig. 4*A*; see *Materials and Methods*). Intrinsic rewards are given to agents internally whenever they encounter a “novel,” “surprising,” or “informative” state. In contrast, extrinsic rewards are received only at the three goal states (*SI Appendix*). Specifically, at each time t, an agent observes state $s_{t}$, evaluates its intrinsic reward value $r_{\text{int},t}$ (e.g., the novelty of state $s_{t}$), and evaluates also its extrinsic reward value $r_{\text{ext},t}$ (which is zero except at the goal states). Intrinsic and extrinsic reward values are then passed to two parallel but separate RL systems, each working with a single reward signal.

The two RL systems use a hybrid algorithm (50, 58) that combines model-based planning (59, 60) and model-free habit formation (61) to estimate one set of Q-values $Q_{\text{ext},t}$ for future extrinsic rewards and another set $Q_{\text{int},t}$ for future intrinsic rewards (20, 37). The next action $a_{t}$ is then sampled from a softmax policy $\pi_{t}$ based on a weighted combination of the Q-values, i.e., $\beta_{\text{int}}Q_{\text{int},t}+\beta_{\text{ext}}Q_{\text{ext},t}$ (Fig. 4*A*; see *SI Appendix*). The amplitudes of the combination weights, *β*_int_ and *β*_ext_, determine the degree of random exploration, while their ratio governs the extent to which intrinsic rewards drive exploration. Hence, to explicitly model the link between exploration and reward optimism, we assumed that these weights depend on the degree of “reward optimism.” To do so, we specified *β*_int_ and *β*_ext_ to i) differ between episode 1 and episodes 2 to 5, and ii) depend, during episodes 2 to 5, on the reward magnitude of $G^{*}$ discovered in episode 1 (*Materials and Methods* and *SI Appendix*). As a result, reward optimism, by design, controls the extent of exploration in our model, regardless of which intrinsic reward drives exploration.

We formulated three different hypotheses for human exploration in the form of three types of intrinsic rewards $r_{\text{int},t}$; all three are representative examples of classes of intrinsic rewards in machine learning (20, 21): i) novelty (13, 14, 37), ii) information gain (17, 19, 62, 63), and iii) surprise (15, 43, 64). Novelty quantifies how infrequent the state $s_{t}$ has been until time t; thus, exploration in novelty-seeking agents is guided toward the least visited states. Information gain quantifies how much the agent updates its belief about the structure of the environment upon observing the transition from the state–action pair $\left(s_{t-1},a_{t-1}\right)$ to state $s_{t}$; thus, exploration in information-gain-seeking agents is guided toward states where the agents’ estimates of the transition probabilities are least certain. Surprise quantifies how unexpected it is to observe state $s_{t}$ after taking action $a_{t-1}$ at state $s_{t-1}$; thus, exploration in surprise-seeking agents is guided toward states with the most stochastic actions.

As a control, we also considered the hypothesis that no explicit intrinsic reward signal is needed to explain human exploratory actions. We formalized this hypothesis in the form of an algorithm that uses no intrinsic reward but incorporates some exploration incentive into the model via optimistic initialization of the Q-values for extrinsic rewards (49). We note that the three algorithms with intrinsic rewards (i.e., novelty-seeking, information-gain-seeking, and surprise-seeking) also enable optimistic initialization; see *SI Appendix* for details.

### Novelty Is the Most Likely Drive of Human Exploration.

To test which algorithm best explains human behavior, we used threefold cross-validation (68): we fitted the parameters of our four algorithms (i.e., novelty-seeking, information-gain-seeking, surprise-seeking, and exploration with no intrinsic reward) to the action choices of two-thirds of the participants by maximizing the likelihood of data given the model parameters (*SI Appendix*). We then evaluated each algorithm’s predictive power by computing the likelihood of the remaining participants’ data under the fitted parameters (*SI Appendix*). For each algorithm, this approach enables us to identify the parameters that provide the closest approximation to human behavior. We emphasize that the only difference between the four algorithms is the type of intrinsic reward used for exploration.

Given the cross-validated likelihood of different algorithms, we used Bayesian model comparison (41, 66) to rank the models (*SI Appendix*). We find that seeking novelty is by far the most probable model for the majority of human participants, followed by seeking information gain as the 2nd most probable model [Fig. 4*B*; model-recovery (67) in *Inset*]. Repeating the model comparison separately for each group of participants yielded the same conclusion (Fig. 4*D*; despite the ∼70% decrease in the sample size). This result shows i) that seeking novelty describes the behavior of human participants better than seeking information gain, seeking surprise, or exploration with no intrinsic reward and ii) that reward optimism mainly influences the extent of the exploration but does not have a strong influence on the exploration strategy. In other words, if we were to summarize the thousands of actions taken by participants into a handful of parameters, our results indicate that the hybrid RL algorithm with a novelty-seeking component would provide the most accurate summary of the data among our candidate models.

**Fig. 5.**
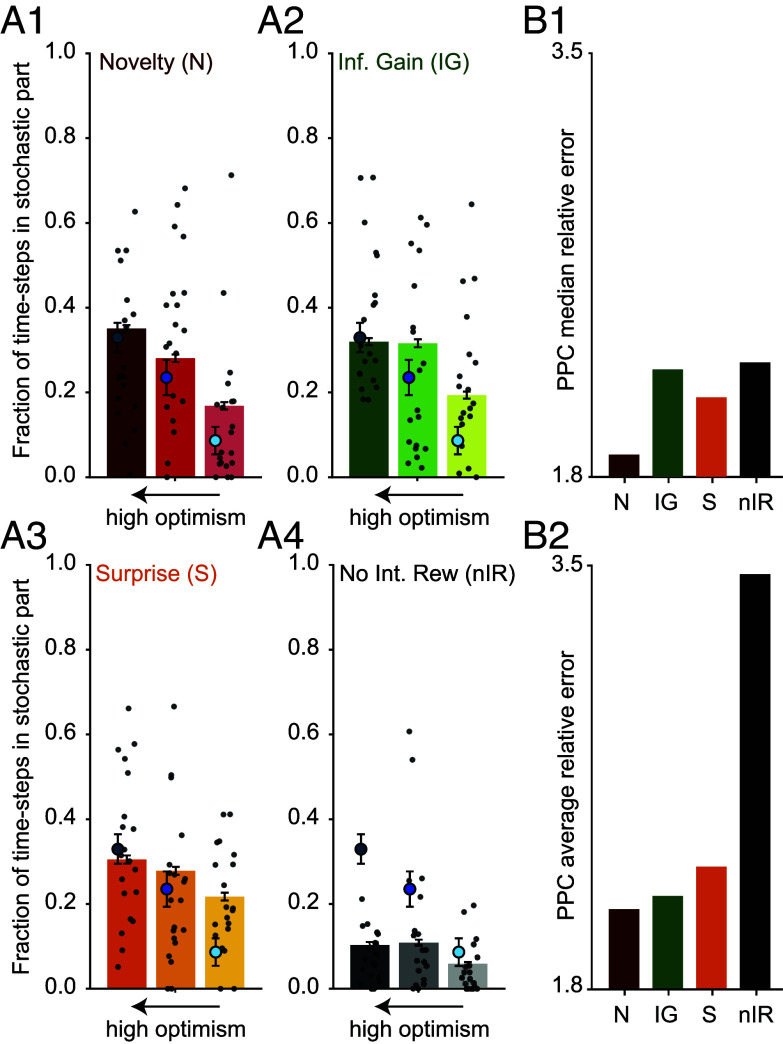
Model-comparison based on PPC. (*A*) Comparing different algorithms with respect to how accurately they replicate the fractions of time-steps spent in the stochastic part during episodes 2 to 5. Blue points show the human behavioral data of Fig. 2*B*2, and the red bars in A1 correspond to data from the simulated agents by novelty-seeking in Fig. 2*E*2. (*B*) Median (*B*1) and average (*B*2) relative error (i.e., absolute difference divided by the SE) of each algorithm in replicating 43 group-level summary statistics of the action choices of human participants. See *SI Appendix* for details and the full list of statistics.

To confirm the results of our model comparison, we next asked how well, if at all, the fitted algorithms could reproduce the statistical properties of the data. To address this, we simulated each of the four algorithms, using their fitted parameters, within our experimental paradigm, i.e., we performed PPC (67, 69). First, the PPC results confirmed that the fitted novelty-seeking reproduces the key qualitative patterns of human behavior (compare Fig. 2*A*–*C* with *D*–*F*). We then went beyond these few patterns and compared 43 summary statistics of human action choices (e.g., the fraction of time-steps spent in the stochastic part; Fig. 5*A*) with those of the simulated agents (see *SI Appendix* for the full list of summary statistics). While several qualitative effects were also approximately reproduced by the surprise-seeking and information-gain-seeking algorithms, novelty-seeking was the most quantitatively accurate in capturing the full statistical structure of human behavior (Fig. 5*B* and *SI Appendix*). These results confirm that the hybrid RL algorithm with a novelty-seeking component best summarizes the key patterns of human behavior—while we emphasize that this summary may be far from perfect (*Discussion*).

Finally, to test the predictive power of novelty-seeking, we quantified its accuracy in predicting individual actions of human participants, i.e., given a participant’s actions until time t, we asked whether novelty-seeking could predict the participant’s action at t+1 (*SI Appendix*). We found a more than 40% cross-validated accuracy rate in episode 1 (Fig. 4*C*; chance level: 33%). As the participants moved through the environment, their behavior became more predictable (i.e., it was determined more strongly by their experience throughout the experiment than by their life experience before the experiment): We observed an increase in the cross-validated accuracy rate for episodes 2 to 5, with an accuracy rate of more than 60% in episode 5. Therefore, novelty-seeking enabled an above-chance prediction of each participant’s actions, even though it had no prior information about the participant.

Taken together, our results provide quantitative and qualitative evidence for novelty as a candidate driver of human exploration in our experiment.

## Discussion

We designed an experimental paradigm to study human goal-directed exploration in multistep stochastic environments with sparse rewards. We made three main observations: i) Human participants who were optimistic about finding higher rewards than those already discovered were persistently attracted to the stochastic part; ii) the extent of attraction to the stochastic part decreased by decreasing the participants’ level of optimism, but it did not vanish even when there was no prospect of finding better rewards than the one already discovered; and iii) this exploratory behavior was explained more accurately by seeking novelty than seeking information gain or surprise.

These three observations are instrumental in addressing the long-standing question of how humans explore their environments (4–6). Specifically, past experimental studies have shown that humans use a combination of random and directed exploration in 1-step or 2-step decision-making tasks (e.g., multiarmed bandits) (22–24, 70–72), and theoretical studies have proposed distinct motivational signals as potential drives of human directed exploratory actions (5, 8, 9, 73, 74). However, despite significant advances (25–27, 29–31, 75–82), it has remained highly debated which motivational signal best explains human exploration (9, 10). The focus of existing studies on 1-step or 2-step decision-making tasks has raised questions about whether our current understanding of human exploration can be generalized to more complex and realistic situations (9, 34–36, 39).

To bridge between exploration in 1-step and multistep tasks, we showed in an earlier study (37) that novelty most accurately explains human exploration in complex but deterministic environments with sparse rewards. Observations (i)–(iii) above provide further evidence for novelty-seeking as the most accurate candidate for human goal-directed exploration even in situations with heterogeneous stochasticity, when seeking novelty is not necessarily optimal. Specifically, after episode 1, participants can reasonably assume that the task is solvable, i.e., if they have succeeded in finding the 2 CHF reward, then they should also be able to find the higher rewards. Hence, the fact that the participants in the 2 CHF group continued the search during episodes 2 to 5 is expected and economically rational, but our results show that they overexplored the stochastic part of the environment—likely due to the use of a suboptimal novelty-based search strategy. Further experimental studies are needed to investigate the implications of our results for other types of human exploratory behavior. In particular, it is a priori unclear whether goal-directed exploration, as studied here, shares some drives and mechanisms with reward-free exploration strategies in, e.g., reactive orienting and passive viewing (79, 83), navigation (84, 85), and noninstrumental decision-making tasks (29, 32, 33).

Our experimental paradigm features complexity across several dimensions, including a large yet unknown number of states, heterogeneous stochasticity, and multiple goal states with differing reward values. While this complexity enabled us to uncover some critical patterns in human exploration, it also necessitated a higher degree of complexity in computational modeling. As a result, our candidate algorithms are considerably more complex than conventional “theory-driven” models in cognitive science (e.g., refs. 24, 50, 77 and 86) and may be better understood as data-summarization tools rather than formal cognitive theories. Specifically, our modeling goal can be seen as identifying the exploration strategy that best summarizes thousands of participant actions using approximately 30 interpretable parameters (*Materials and Methods*). In this regard, our modeling approach strikes a practical balance between flexibility and interpretability—sufficiently rich to account for behavior in our multistep, stochastic environment, yet structured enough to offer insight into the underlying mechanisms of exploration. However, in line with the recent success of complex function approximators in cognitive modeling (87–89), we also acknowledge that we cannot confidently claim that our fitted hybrid model with novelty-seeking reflects the true cognitive process underlying human exploration. This underscores the importance of complementary modeling approaches in future studies.

Our results appear to contradict the long-standing belief that humans are not prone to the noisy TV problem (1, 46, 48). It is important to note, however, that the stochasticity in our environment differs from passively watching a noisy, gray-flickering TV screen. Rather, participants could take actions in our experiment, akin to switching between TV channels, each offering novel and variable contents; this, in fact, is closely similar to a recent implementation of the noisy TV problem in machine learning (43). In this respect, our experimental paradigm resembles modern social media platforms, where users spend extended periods engaging with “endless scrolling” to discover new videos (90, 91)—despite the availability of alternative activities with clearer extrinsic rewards. This user behavior is analogous to the behavior of participants in the 4 CHF group, who continued to explore the stochastic part despite knowing the path to the most rewarding goal state. While we have focused primarily on the influence of reward optimism in explaining this overexploration, other factors—such as time or action costs—could similarly modulate exploratory behavior. However, introducing such elements would also bring additional confounds and shift the task away from the noisy TV formulation in the machine learning community. Systematically incorporating such costs remains an interesting direction for future work.

Finally, we note that notions of novelty, surprise, and information gain as scientific terms often refer to different precise mathematical definitions (64, 92)—across a broad set of applications in neuroscience (37, 93, 94), psychology (95–97), and machine learning (20, 21, 48). Our results in this paper are based on the specific mathematical formulations that we have chosen (*Materials and Methods*), but we expect our conclusions to be invariant to the precise choice of definitions as long as i) novelty quantifies infrequency of states (37) as, for example, defined with density models in machine learning (13, 14, 98); ii) surprise quantifies mismatches between observations and an agent’s expectations, where the expectations are made based on the previous state–action pair, including all measures of prediction surprise (64) and typical measures of prediction error in machine learning (15, 43); and iii) information gain quantifies improvements in the agents’ world-model and vanishes with the accumulation of experience, which includes Bayesian (93) and Postdictive surprise (94), measures of disagreement and progress-rate in machine learning (17–19, 44, 99), and optimal exploration bonuses in RL theory (100, 101).

In conclusion, our results show i) that human decision-making is influenced by an interplay of intrinsic and extrinsic rewards, controlled by reward optimism, and ii) that novelty-seeking RL algorithms are strong candidates for modeling this interplay.

## Materials and Methods

### Ethics Statement.

The data for human experiment were collected under CE 164/2014, and the protocol was approved by the “Commission cantonale d’éthique de la recherche sur l’être humain.” All participants were informed that they could quit the experiment at any time and signed a written informed consent. All procedures complied with the Declaration of Helsinki (except for preregistration).

### Participants.

Sixty-three participants joined the experiment through voluntary sampling—in response to advertisements placed on the EPFL campus website. Data from 6 participants were removed (see below), and the data from the remaining 57 participants (27 female, mean age 24.1±4.1 y) were included in the analyses. The sample size was chosen to be within the typical range of in-lab experiments (e.g., refs. 61, 86, and 102) and to ensure that, for each goal condition, we had twice as many participants as in our previous study on multistep decision-making (37). All participants were naive to the purpose of the experiment and had normal or corrected-to-normal visual acuity. The experiment was scripted in MATLAB using the Psychophysics Toolbox (103).

### Experimental Procedure.

Before starting the experiment, participants played a demo to familiarize themselves with navigating a simple environment with a different structure from that of the main experiment. They were explicitly instructed on how action selection works and how they could transition between images by selecting different actions. Participants were given a written instruction (*SI Appendix*) but were also debriefed in person. They were then informed that both the environment’s structure and the state images were different in the main experiment from those in the demo. Importantly, they were explicitly told that “[We] cannot tell whether the experiment you are going to do is deterministic or stochastic” (*SI Appendix*). Additionally, the participants were informed that there were three goal states, and they need to find any of the 3 goal states 5 times. They were shown the 3 goal images and informed that each image had a different reward value of 2 CHF, 3 CHF, or 4 CHF. Specifically, they were given an example that “if you find [the 2 CHF goal] twice, [the 3 CHF goal] once, and [the 4 CHF goal] twice, then you will be paid 2×2+1×3+2×4=15 CHF”; see *SI Appendix* for how this information was incorporated into the RL algorithms. At each trial, participants were presented with an image (state) and three gray disks below the image (Fig. 1*C*). Clicking on a disk (action) led participants to a subsequent image, which was chosen based on the underlying graph of the environment in Fig. 1 *A* and *B* (which was unknown to the participants). Participants clicked through the environment until they found one of the goal states, which finished an episode (Fig. 1*C*).

The assignment of images to states and disks to actions was random but kept fixed throughout the experiment and identical for all participants (Fig. 1*C*2). Exceptionally, we did not make the assignment for the actions in state 4 before the start of the experiment. Rather, for each participant, we assigned the disk that was chosen in the 1st encounter of state 4 to the stochastic action and the other two disks randomly to the bad and progressing actions, respectively (Fig. 1*A*). With this assignment, we ensured that all human participants would visit the stochastic part at least once during episode 1. The same protocol was used for simulated RL agents. Additionally, to ensure that participants would not get lost in the stochastic part, we used the same assignment for the “escape action” in all stochastic states (i.e., the action that took participants from stochastic states to state 4 in Fig. 1*B*).

Before the start of the experiment, we randomly assigned the different goal images (corresponding to the three reward values) to different goal states $G^{*}$, $G_{1}$, and $G_{2}$, separately for each participant (Fig. 1*D*). The image and, hence, the reward value were then kept fixed throughout the experiment. In other words, we randomly assigned different participants to different environments with the same structure but different assignments of reward values. We, therefore, ended up with three groups of participants: 23 in the 2 CHF group, 20 in the 3 CHF group, and 20 in the 4 CHF group (Fig. 1*D*). The probability of encountering a goal state other than $G^{*}$ was controlled by the parameters ε. We considered ε to be around machine precision $10^{-8}$, so we have ${\left(1-\varepsilon \right)}^{5\times 63}\approx 1-10^{-5}\approx 1$, meaning that all 63 participants would be taken almost surely to the goal state $G^{*}$ in all five episodes.

Two participants (in the 2 CHF group) did not finish the experiment, and four participants (1 in the 3 CHF group and 3 in the 4 CHF group) took more than 3 times the group-average number of actions in episodes 2 to 5 to finish the experiment. We considered this as a sign of being nonattentive and removed these 6 participants from further analyses.

At the end of the experiment, participants were given a paper with the pseudorandomly placed images of progressing states (1 to 6), trap states (7 to 8), one stochastic state (S-44), a new image (X) that did not belong to the 58 states of the environment, and the three goal states. Participants were asked to “draw the transitions between images” and were told they “can add anything [they] want.” Some participants had not reported the directionality of transitions. Hence, we only analyzed how many participants had drawn a link between every pair of states, independently of the link’s direction (Fig. 3). Moreover, most participants had ignored the transitions to the goal states, so we excluded the goal states from the analysis. To further simplify analyses, we did not distinguish between different trap states when counting the connections from nontrap states to the trap states. As a result, there were 1+9×8/2=37 possible links to draw (the extra 1 belongs to the connection between the two trap states), but there were only 13 links in the ground truth (Fig. 3*A*, *Inset*). Accordingly, we defined the reconstruction score in Fig. 3 as the ratio of correctly reconstructed links (out of 13) minus the ratio of incorrectly reconstructed links (out of 24). This limits the reconstruction score to the range [−1,1] with the chance level at 0.

### Statistical Tests.

The correction for multiple hypotheses testing was done by controlling the false discovery rate at 0.05 (54) over all 13 null hypotheses that are presented in Figs. 2 and 3 (*P*-value threshold: 0.046). All Bayes Factors (abbreviated BF in the figures) were evaluated using the Schwartz approximation (53) to avoid any assumptions on the prior distribution.

### Intrinsically Motivated RL as a Model of Human Behavior.

We used ideas from nonparametric Bayesian inference (104) to design an intrinsically motivated RL agent that operates in an unknown and expanding state space (see also refs. 62 and 105 for alternatives). Below, we present three key ingredients of the model that are essential for interpreting our results; the algorithmic details and derivations are presented in *SI Appendix*.

The first ingredient is the dual value streams to simultaneously learn extrinsic and intrinsic Q-values (visualized as parallel pathways in Fig. 4*A*). The extrinsic Q-value $Q_{\text{ext},t}\left(s,a\right)$ estimates the expected future extrinsic reward of taking action a in state s, while $Q_{\text{int},t}\left(s,a\right)$ represents the analogous value for the intrinsic reward (49). All candidate algorithms share this architecture and receive the same extrinsic reward signal $r_{\text{ext},t}$, but they differ in the signal they treat as intrinsically rewarding. We present the different types of intrinsic rewards in the next section.

The second key ingredient is the nonparametric estimation of the environment’s transition probability $p_{t}$ and the empirical state frequency $p_{t}$. The quantity $p_{t}\left(s^{\prime }|s,a\right)$ represents the agent’s estimate of the probability of transitioning to $s^{\prime }$ after taking action a in state s. The quantity $p_{t}\left(s^{\prime }\right)$, on the other hand, reflects how frequently $s^{\prime }$ has been visited so far—independently of the previous state s or action a. These two estimates underlie fundamental distinctions between surprise and novelty (see ref. 106): The transition probability $p_{t}\left(s^{\prime }|s,a\right)$ captures how “unpredictable” or “unexpected” $s^{\prime }$ is, conditioned on s and a, whereas the empirical frequency $p_{t}\left(s^{\prime }\right)$ captures the “relative familiarity” of $s^{\prime }$ compared to other states. This distinction forms the basis for the intrinsic reward functions described in the next section.

The final key ingredient is the hybrid softmax policy, whose simplified form is given by (see *SI Appendix* for the full version)

[1] $$\begin{array}{c} \pi_{t}(a|s)\propto \exp[\beta_{\text{ext}}Q_{\text{ext},t}(s,a)+\beta_{\text{int}}Q_{\text{int},t}(s,a)]. \end{array}$$

The inverse-temperature parameters *β*_ext_ and *β*_int_ both control the stochasticity of action selection (i.e., the degree of random exploration) and determine the weighting of intrinsic vs. extrinsic value (i.e., the degree of exploration driven by intrinsic reward). After the goal $G^{*}$ is discovered, at the end of episode 1, we allowed *β*_ext_ and *β*_int_, for all models, to vary across goal conditions (2,3, and 4 CHF) but fixed across participants within each group. This naturally captures the experimentally induced “reward optimism” that modulates the balance between exploration and exploitation.

With all components combined, the algorithms with intrinsic rewards (i.e., novelty-seeking, surprise-seeking, and information-gain-seeking) had 27 free parameters. Removing the branch for intrinsic rewards yields our fourth algorithm (nRI in Figs. 4 and 5), which had 19 parameters. Models were compared according to their test log likelihood, evaluated by stratified threefold cross-validation. See *SI Appendix* for details.

### Different Types of Intrinsic Rewards.

Our candidate algorithms (represented by different colors in Figs. 4 and 5) share the same modeling architecture described above, but differ in their definitions of intrinsic reward. Below, we present a compact formulation of each intrinsic reward; technical details are presented in *SI Appendix*.

For an agent seeking novelty (red in Figs. 4 and 5), we defined the intrinsic reward $r_{\text{int},t}$ as the novelty of state $s^{\left(t\right)}$, i.e.,

[2] $$\begin{array}{c} r_{\text{int},t}:=-\log{\tilde{p}}_{t-1}(s_{t}). \end{array}$$

According to this definition, more frequently visited states receive lower novelty values—and therefore lower intrinsic rewards. For an agent seeking surprise (orange in Figs. 4 and 5), the intrinsic reward $r_{\text{int},t}$ was defined as the Shannon surprise (a.k.a. surprisal) of observing $s_{t}$ conditioned on $s_{t-1}$ and $a_{t-1}$, i.e.,

[3] $$\begin{array}{c} r_{\text{int},t}:=-\log p_{t-1}\left(s_{t}|s_{t-1},a_{t-1}\right). \end{array}$$

With this definition, the expected (i.e., averaged over $s_{t}$) intrinsic reward of taking action a at state s corresponds to the entropy of the distribution $p_{t-1}\left(s^{\prime }|s,a\right)$ (107). Thus, surprise-seeking can also be interpreted as seeking (aleatoric) uncertainty. On the other hand, for an agent seeking information gain (green in Figs. 4 and 5), we defined the intrinsic reward as

[4] $$\begin{array}{c} r_{\text{int},t}:=\;{\text{D}}_{\text{KL}}\left(p_{t-1}\;\|\;p_{t}\right), \end{array}$$

where ${\text{D}}_{\text{KL}}$ denotes the Kullback–Leibler divergence (107), and $p_{t}$ is the updated estimate of transition probabilities after observing the transition $\left(s_{t-1},a_{t-1}\right)\to s_{t}$. While this definition of information gain can also be interpreted as a form of surprise—specifically, “postdictive surprise” (94)—its behavior substantially differs from that of the Shannon surprise used in surprise-seeking agents; see ref. (64) for a detailed comparison and *SI Appendix* for simulations. As a final alternative (black in Fig. 4-5), we considered agents with no intrinsic reward, i.e.,

[5] $$\begin{array}{c} r_{\text{int},t}:=0. \end{array}$$

Exploration in these agents is purely driven by optimistic initialization (*SI Appendix*).

## Supplementary Material

Appendix 01 (PDF)

## Data Availability

Behavioral choices’ data have been deposited in GitHub (https://github.com/modirshanechi/ComplexEnvExploration-Modirshanechi2025) (108) and Zenodo (https://doi.org/10.5281/zenodo.16962408) (109).
