# Supplementary material for: Novelty as a drive of human exploration in complex stochastic environments
Source: Proc Natl Acad Sci U S A. 2025 Sep 25;122(39):e2502193122. doi: 10.1073/pnas.2502193122 (PMC12501151; doi:10.1073/pnas.2502193122)
Supplement: Supplementary file 1 — Appendix 01 (PDF) [file pnas.2502193122.sapp.pdf]

Supplementary materials to
"Novelty as a drive of human exploration in complex stochastic environments"

Modirshanechi*, Lin, Xu, Herzog, and Gerstner

*alireza.modirshanechi@helmholtz-munich.de

Contents

1 Supplementary Methods: Task instruction **2**

2 Supplementary Methods: Computational modeling **3**
2.1 Intrinsically motivated RL as a computational model of human behavior 3
2.2 Informing RL agents of different goal states 6
2.3 The four algorithms compared in the main text 8
2.3.1 Novelty-seeking 8
2.3.2 Information-gain-seeking 9
2.3.3 Surprise-seeking 9
2.3.4 Exploration with no intrinsic reward 9
2.4 Model-fitting and model-comparison 9
2.5 Posterior predictive checks and model-recovery 11

3 Supplementary Results **12**
3.1 Different intrinsic rewards react differently to stochasticity 12
3.2 Detailed summary statistics for posterior predictive checks 15
3.3 Posterior predictive checks with no exclusion criterion 16
3.4 Model-selection with uniformly leaking counts 17

4 Supplementary Methods: Derivations and theoretical analyses **18**
4.1 Model-building in an environment of unknown size 18
4.1.1 Time-dependent base distribution as the expected prior 18
4.1.2 Derivation of the world-model 18
4.2 Prioritized sweeping for updating the MB Q-values 20
4.3 Derivation of information gain 21
4.4 Seeking surprise or information gain in deterministic environments 22
4.5 Analysis of the MB optimistic initialization in episode 2 24
4.6 Optimistic initialization in tandem with intrinsic rewards 26

5 Supplementary Methods: Algorithmic implementation **27**
5.1 Initialization 27
5.2 Pseudocode 27

References **31**

Note that Figure 1-Figure 5 always refer to the figures in the main text.

Supplementary figures are indexed as SFigure 1, SFigure 2, etc.

1 Supplementary Methods: Task instruction

Participants first played a demo to familiarize themselves with navigating a simple environment with a different structure than that of the main experiment. During the demo, they were briefed on how the action selection worked and how they could transition between images by selecting different actions. Then, they were given a written instruction with the following information:

1. ‘Different from the demo, at the beginning of this experiment, I will not show you all the images that you will see in the experiment.’
2. ‘We cannot tell whether the experiment you are going to do is deterministic or stochastic.’
3. ‘There are three types of rewards in this experiment:
   - The image of a 10-Euro bill: every time you find this image, you will receive 2 CHF
   - The image of a 50-Euro bill: every time you find this image, you will receive 3 CHF
   - The image of a 100-Euro bill: every time you find this image, you will receive 4 CHF’
4. ‘During the experiment, you need to find the rewards 5 times. You will be paid based on which rewards you find. For example, if you find the ‘10 Euro bill’ twice, ‘50 Euro bill’ once, and ‘100 Euro bill’ twice, you will be paid $2\times2 + 3\times1 + 4\times2 = 15$ CHF.’

2 Supplementary Methods: Computational modeling

2.1 Intrinsically motivated RL as a computational model of human behavior

We used ideas from non-parametric Bayesian inference [1] to design an intrinsically motivated RL algorithm for environments where the total number of states is unknown. We present the final results here, the derivations and some theoretical analysis in Section 4, and the pseudo-code in Section 5. A summary of the mathematical notation is provided in Table 1.

We indicate the sequence of actions and states until time t by $s_{1:t}$ and $a_{1:t}$, respectively, and define the **set of all known states** at time t as

$$\mathcal{S}^{(t)} = \Big\{ s : \exists t' \in \{1, ..., t\} \text{ s.t. } s = s_{t'} \Big\} \cup \{\tilde{G}_0, \tilde{G}_1, \tilde{G}_2\}, \tag{S1}$$

where $\tilde{G}_i$s represent our three different goal states—$\tilde{G}_0$ corresponds to the 2 CHF goal, $\tilde{G}_1$ to the 3 CHF goal, and $\tilde{G}_2$ to the 4 CHF goal. Note that $\{\tilde{G}_0, \tilde{G}_1, \tilde{G}_2\}$ represents the images of the goal states and not their locations G^*, G_1, and G_2; the assignment of images to locations is unknown to the model. Hence, starting with $t = 0$, the algorithm incorporates information about the existence of multiple goal states in the environment. In a more general setting, $\{\tilde{G}_0, \tilde{G}_1, \tilde{G}_2\}$ should be replaced by the set of all states whose images were shown to participants before the experiment. After a transition to state $s_{t+1} = s'$ resulting from taking action $a_t = a \in$ {left, middle, right} (i.e., representing disk positions in Figure 1C) at state $s_t = s$, the reward functions R_{ext} and $R_{\text{int},t}$ evaluate the reward values $r_{\text{ext},t+1}$ and $r_{\text{int},t+1}$. We define the **extrinsic reward function** R_{ext} as

$$R_{\text{ext}}(s, a \to s') = \delta_{s',\tilde{G}_0} + r_1^* \delta_{s',\tilde{G}_1} + r_2^* \delta_{s',\tilde{G}_2}, \tag{S2}$$

where δ is the Kronecker delta function, and we assume (without loss of generality) a subjective extrinsic reward value of 1 for $\tilde{G}_0$ (2 CHF goal) and subjective extrinsic reward values of $r_1^* \geq 1$ and $r_2^* \geq 1$ for $\tilde{G}_1$ and $\tilde{G}_2$, respectively. The prior information of human participants about the difference in the monetary reward values of different goal states can be modeled in simulated RL agents by varying r_1^* and r_2^* (resulting in the exploratory component of reward-seeking via optimistic initialization; see Section 2.2). We discuss $R_{\text{int},t}$ in Section 2.3.

As a general choice for the RL algorithm in Figure 4A, we considered a hybrid of model-based and model-free policy [2–5]. The **model-free (MF) component** uses the sequence of states $s_{1:t}$, actions $a_{1:t}$, extrinsic rewards $r_{\text{ext},1:t}$, and intrinsic rewards $r_{\text{int},1:t}$ (in the two parallel branches in Figure 4A) and estimates the extrinsic and intrinsic Q-values $Q^{(t)}_{\text{MF,ext}}$ and $Q^{(t)}_{\text{MF,int}}$, respectively. Traditionally, MF algorithms do not need knowledge of the total number of states [6]; thus, the MF component of our algorithm remains similar to that of previous studies [2, 7]: At the beginning of episode 1, Q-values are initialized at $Q^{(0)}_{\text{MF,ext}}$ and $Q^{(0)}_{\text{MF,int}}$. Then, the estimates are updated recursively after each new observation. After the transition $(s_t, a_t) \to s_{t+1}$, the agent computes extrinsic and intrinsic reward prediction errors $RPE_{\text{ext},t+1}$ and $RPE_{\text{int},t+1}$, respectively:

$$\begin{aligned} RPE_{\text{ext},t+1} &= r_{\text{ext},t+1} + \gamma_{\text{ext}} V^{(t)}_{\text{MF,ext}}(s_{t+1}) - Q^{(t)}_{\text{MF,ext}}(s_t, a_t) \\ RPE_{\text{int},t+1} &= r_{\text{int},t+1} + \gamma_{\text{int}} V^{(t)}_{\text{MF,int}}(s_{t+1}) - Q^{(t)}_{\text{MF,int}}(s_t, a_t), \end{aligned} \tag{S3}$$

where γ_{ext} and $\gamma_{\text{int}} \in [0, 1)$ are the discount factors for extrinsic and intrinsic reward seeking, respectively, and $V^{(t)}_{\text{MF,ext}}(s_{t+1}) = \max_{a'} Q^{(t)}_{\text{MF,ext}}(s_{t+1}, a')$ and $V^{(t)}_{\text{MF,int}}(s_{t+1}) = \max_{a'} Q^{(t)}_{\text{MF,int}}(s_{t+1}, a')$ are the extrinsic and intrinsic V-values of the state s_{t+1}, respectively. We used two separate eligibility traces [6, 7] for the update of Q-values, one for extrinsic reward $e^{(t)}_{\text{ext}}$ and one for intrinsic reward $e^{(t)}_{\text{int}}$, both initialized at zero at the beginning of each episode. The update rules for the eligibility traces after taking action a_t at state

Symbol	Description	
$\tilde{G}_i$	Image-label of goal state i $(2, 3, 4$ CHF).	(Equation S1)
$\mathcal{S}^{(t)}$	Set of known states up to t, including $\tilde{G}_i$s.	(Equation S1)
G^*	Actual goal state discovered in Episode 1.	(Alg. 1)
$R_{\text{ext}}(s, a \to s')$	Extrinsic reward function.	(Equation S2)
$R_{\text{int},t}(s, a \to s')$	Intrinsic reward function.	(Sec. 2.3)
$Q^{(t)}_{\text{MB/MF, ext/int}}$	State-action pair values.	(Equation S5; Alg. 4)
$V^{(t)}_{\text{MB/MF, ext/int}}$	State-values.	(Equation S3; Alg. 4)
$RPE_{\text{ext/int},t}$	Reward prediction errors.	(Equation S3)
$e^{(t)}_{\text{ext/int}}$	Eligibility traces.	(Equation S4)
$\tilde{C}^{(t+1)}_{s,a,s'}$	(Leaky) count of transitions $(s, a) \to s'$.	(Equation S6)
$p^{(t)}(s' \mid s, a) = \hat{\theta}^{(t)}_{s,a}(s')$	Estimated transition probability.	(Equation S7)
$p^{(t)}_f(s)$	Empirical frequency (relative familiarity) of s; same as $\tilde{p}_t$ in the main text.	(Equation S16)
$\pi_t(a\|s)$	Softmax policy combining all values.	(Equation S8)
r^*_1 & r^*_2	Subjective extrinsic values for $\tilde{G}_1$ & $\tilde{G}_2$.	(Equation S2)
γ_{ext} & γ_{int}	Discount factors.	(Equation S3; Alg. 4)
λ_{ext} & λ_{int}	Decay factor of eligibility traces.	(Equation S4)
ρ	Learning rate for MF updates.	(Equation S5)
κ	Leak parameter for transition counts.	(Equation S6)
ϵ_{known} & ϵ_{new}	Prior counts for the known & new states.	(Equation S7)
$T_{PS,\text{ext/int}}$	Number of prioritized sweeping iterations.	(Alg. 4)
$\beta^{(1)}_{\text{MB/MF, ext/int}}$	Policy inverse-temperatures in Episode 1.	(Equation S8; Alg. 1)
$\beta^{(2,r)}_{\text{MB/MF, ext/int}}$	Policy inverse-temperatures in Episodes 2-5, depending on the reward value r of G^*.	(Equation S8; Alg. 1)
$b(a)$	Action-bias term, with $b(\text{left}) = 0$.	(Equation S8)

Table 1: Main notation and definitions.

s_t is

$$e^{(t+1)}_{\text{ext}}(s,a) = \begin{cases} 1 & \text{if} \quad s = s_t, a = a_t \\ \gamma_{\text{ext}}\lambda_{\text{ext}} e^{(t)}_{\text{ext}}(s,a) & \text{otherwise} \end{cases}$$
$$e^{(t+1)}_{\text{int}}(s,a) = \begin{cases} 1 & \text{if} \quad s = s_t, a = a_t \\ \gamma_{\text{int}}\lambda_{\text{int}} e^{(t)}_{\text{int}}(s,a) & \text{otherwise}\,, \end{cases} \tag{S4}$$

where γ_{ext} and γ_{int} are the discount factors defined above, and λ_{ext} and $\lambda_{\text{int}} \in [0, 1]$ are the decay factors

of the eligibility traces for the extrinsic and intrinsic rewards, respectively. The update rule for the MF Q-values is then given by

$$\Delta Q_{\mathrm{MF}}^{(t+1)}(s,a) = \rho e_{t+1}(s,a) RPE_{t+1}, \tag{S5}$$

where e_{t+1} is the eligibility trace (i.e., either $e_{\mathrm{ext}}^{(t+1)}$ or $e_{\mathrm{int}}^{(t+1)}$), RPE_{t+1} is the reward prediction error (i.e., either $RPE_{\mathrm{ext},t+1}$ or $RPE_{\mathrm{int},t+1}$), and $\rho \in [0,1)$ is the learning rate.

In parallel, the **model-based (MB) component** builds a world-model that summarizes the structure of the environment by estimating the probability $p^{(t)}(s'|s,a)$ of the transition $(s,a) \to s'$. We assumed that the agent takes a non-parametric approach: We used Dirichlet Processes to define the agent's estimate $p^{(t)}(s'|s,a)$ as the expected probability of the transition $(s,a) \to s'$, conditioned on $a_{1:t}$ and $s_{1:t}$. While the derivation of this Bayesian estimate requires a number of technical steps (presented in Section 4.1), the final estimate of $p^{(t)}(s'|s,a)$ has a straightforward interpretation and a simple implementation. Specifically, to make the Bayesian estimate, the agent only needs to count the transition $(s,a) \to s'$ recursively, using a leaky integrator [8, 9]:

$$\tilde{C}_{s,a,s'}^{(t+1)} = \begin{cases} \kappa \tilde{C}_{s,a,s'}^{(t)} + \delta_{s',s_{t+1}} & \text{if } s = s_t\,,\ a = a_t \\ \tilde{C}_{s,a,s'}^{(t)} & \text{otherwise,} \end{cases} \tag{S6}$$

where δ is the Kronecker delta function, $\tilde{C}_{s,a,s'}^{(0)} = 0$, and $\kappa \in [0,1]$ is the leak parameter and accounts for imperfect, forgetful model-building in humans; see Section 3.4 for an alternative approach. If $\kappa = 1$, then $\tilde{C}_{s,a,s'}^{(t+1)}$ is the exact count of transition $(s,a) \to s'$. For $\kappa < 1$, we refer to $\tilde{C}_{s,a,s'}^{(t+1)}$ as a leaky count. These leaky counts are used to estimate the transition probabilities

$$p^{(t)}(s'|s,a) = \begin{cases} \frac{\epsilon_{\mathrm{known}} + \tilde{C}_{s,a,s'}^{(t)}}{\epsilon_{\mathrm{new}} + \epsilon_{\mathrm{known}} |\mathcal{S}^{(t)}| + \tilde{C}_{s,a}^{(t)}} & \text{if } \quad s' \in \mathcal{S}^{(t)}\,, \\ \frac{\epsilon_{\mathrm{new}}}{\epsilon_{\mathrm{new}} + \epsilon_{\mathrm{known}} |\mathcal{S}^{(t)}| + \tilde{C}_{s,a}^{(t)}} & \text{if } \quad s' = s_{\mathrm{new}}\,, \end{cases} \tag{S7}$$

where $\tilde{C}_{s,a}^{(t)} = \sum_{s'} \tilde{C}_{s,a,s'}^{(t)}$ is the leaky count of taking action a at state s, $\epsilon_{\mathrm{known}} \in \mathbb{R}^+$ is a free parameter for the prior pseudo-count of transition to a known state (i.e., states in $\mathcal{S}^{(t)}$), and $\epsilon_{\mathrm{new}} \in \mathbb{R}^+$ is a free parameter for the prior pseudo-count of transition to a new state (i.e., states not in $\mathcal{S}^{(t)}$). Choosing $\epsilon_{\mathrm{new}} = 0$ is equivalent to assuming there is no unknown state in the environment, for which the estimate in Equation S7 is reduced to the classic Bayesian estimate of transition probabilities in bounded discrete environments [2, 3]. See Section 4 for derivations and further interpretation.

The transition probabilities are then used in a novel variant of prioritized sweeping [6, 10] adapted to deal with an unknown number of states. The prioritized sweeping algorithm was chosen primarily for practical reasons and implementation efficiency. The algorithm evaluates a pair of Q-values, i.e., $Q_{\mathrm{MB,ext}}^{(t)}$ for extrinsic and $Q_{\mathrm{MB,int}}^{(t)}$ for intrinsic rewards, by iterating over the corresponding Bellman equations [6] for $T_{PS,\mathrm{ext}}$ and $T_{PS,\mathrm{int}}$ iterations, respectively. See Algorithm 4 for details.

Finally, we considered **a softmax action policy** [6]: The probability of taking action a in state s at time t is

$$\begin{aligned} \pi_t(a|s) \propto \exp \Big[& \beta_{\mathrm{MB,ext}} Q_{\mathrm{MB,ext}}^{(t)}(s,a) + \beta_{\mathrm{MF,ext}} Q_{\mathrm{MF,ext}}^{(t)}(s,a) + \\ & \beta_{\mathrm{MB,int}} Q_{\mathrm{MB,int}}^{(t)}(s,a) + \beta_{\mathrm{MF,int}} Q_{\mathrm{MF,int}}^{(t)}(s,a) + b(a) \Big], \end{aligned} \tag{S8}$$

where $\beta_{\mathrm{MB,ext}} \in \mathbb{R}^+$, $\beta_{\mathrm{MF,ext}} \in \mathbb{R}^+$, $\beta_{\mathrm{MB,int}} \in \mathbb{R}^+$, and $\beta_{\mathrm{MF,int}} \in \mathbb{R}^+$ are free parameters (i.e., inverse temperature parameters of the softmax policy) expressing the contribution of each Q-value to action-selection, and $b(a)$ captures the general bias of the agent for taking the particular action a (e.g., left grey disk in Figure 1C) independently of the state s. Without loss of generality, we assumed $b(\text{left}) = 0$ and

considered $b(\text{middle}) \in \mathbb{R}$ and $b(\text{right}) \in \mathbb{R}$ as free parameters.

In general, the contribution of seeking extrinsic reward and seeking intrinsic reward and the MB and MF branches to action selection depends on different factors, including time passed since the beginning of the experiment [4, 11], cognitive load [12], and whether the location of reward is known [2]. Here, we make a simplistic assumption that these contributions (expressed as the 4 inverse temperature parameters) depend only on reward optimism:

- Episode 1: Before finding the goal state, we considered $\beta_{\text{MB,ext}} = \beta^{(1)}_{\text{MB,ext}}$, $\beta_{\text{MF,ext}} = \beta^{(1)}_{\text{MF,ext}}$, $\beta_{\text{MB,int}} = \beta^{(1)}_{\text{MB,int}}$, and $\beta_{\text{MF,int}} = \beta^{(1)}_{\text{MF,int}}$ as four independent free parameters.
- Episodes 2-5: After finding the goal G^*, we considered $\beta_{\text{MB,ext}} = \beta^{(2,r)}_{\text{MB,ext}}$, $\beta_{\text{MF,ext}} = \beta^{(2,r)}_{\text{MF,ext}}$, $\beta_{\text{MB,int}} = \beta^{(2,r)}_{\text{MB,int}}$, and $\beta_{\text{MF,int}} = \beta^{(2,r)}_{\text{MF,int}}$, where r is either 2 CHF, 3 CHF, or 4CHF, resulting in $3 \times 4 = 12$ free parameters.

Summary of free parameters: The full algorithm has 14 main parameters (capturing initialization and learning dynamics)

$$\Phi^{(\text{main})} = \{r_1^*, r_2^*, Q^{(0)}_{\text{MF,ext}}, Q^{(0)}_{\text{MF,int}}, \gamma_{\text{ext}}, \gamma_{\text{int}}, \lambda_{\text{ext}}, \lambda_{\text{int}}, \rho, \kappa, \epsilon_{\text{new}}, \epsilon_{\text{known}}, T_{PS,\text{ext}}, T_{PS,\text{int}}\}, \tag{S9}$$

16 inverse temperature parameters (capturing the randomness in decision-making and the balance of seeking intrinsic versus extrinsic rewards)

$$\Phi^{(\beta)} = \{\beta^{(1)}_{\text{MB,ext}}, \beta^{(1)}_{\text{MB,int}}, \beta^{(1)}_{\text{MF,ext}}, \beta^{(1)}_{\text{MF,int}}\} \cup \{\beta^{(2,r)}_{\text{MB,ext}}, \beta^{(2,r)}_{\text{MB,int}}, \beta^{(2,r)}_{\text{MF,ext}}, \beta^{(2,r)}_{\text{MF,int}}\}_{r\in\{2,3,4\text{CHF}\}}, \tag{S10}$$

and 2 bias parameters

$$\Phi^{(b)} = \{b(\text{middle}), b(\text{right})\}. \tag{S11}$$

We denote the set of all parameters by

$$\Phi = \{\Phi^{(\text{main})}, \Phi^{(\beta)}, \Phi^{(b)}\} \tag{S12}$$

We note that *not* all these parameters were fitted for all algorithms (see Section 2.3).

2.2 Informing RL agents of different goal states

Human participants were informed that the environment had different goal states with different monetary reward values. This information was intended to incentivize exploration after finding the likely goal state G^* at the end of episode 1. We used three mechanisms to incorporate this information into the RL algorithm described above (Section 2.1). Our main focus throughout the paper was on the first mechanism that assigns different values to $\beta_{\text{MB,ext}}$, $\beta_{\text{MF,ext}}$, $\beta_{\text{MB,int}}$, and $\beta_{\text{MF,int}}$ (see Equation S8) depending on the reward value of G^*; this makes **the relative importance of intrinsic rewards** explicitly depend on the difference between the reward value of the discovered goal r_{G^*} and the known reward values r_1^* and r_2^* of the other goal states (Equation S2).

The other two mechanisms are the **model-based optimistic initialization** and **model-free optimistic initialization**. Exploration in the nIR algorithm in Figure 4 is solely directed via these mechanisms (see Section 2.3). In this section, we discuss how these mechanisms balance exploration versus exploitation.

Model-based optimistic initialization. MB optimistic initialization is an explicit approach to model reward-optimism through designing the world-model. The MB branch finds the extrinsic Q-values $Q^{(t)}_{\text{MB,ext}}$

by (approximately) solving the Bellman equations

$$Q^{(t)}_{\text{MB,ext}}(s,a) = \bar{R}^{(t)}_{\text{ext}}(s,a) + \gamma_{\text{ext}} \sum_{s'} p^{(t)}(s'|s,a) \max_{a'} Q^{(t)}_{\text{MB,ext}}(s',a'), \tag{S13}$$

where $p^{(t)}(s'|s,a)$ is the estimated transition probability in Equation S7, and

$$\begin{aligned}\bar{R}^{(t)}_{\text{ext}}(s,a) &= \sum_{s'} p^{(t)}(s'|s,a) R_{\text{ext}}(s,a \to s') \\ &= p^{(t)}(\tilde{G}_0|s,a) + r_1^* p^{(t)}(\tilde{G}_1|s,a) + r_2^* p^{(t)}(\tilde{G}_2|s,a)\end{aligned} \tag{S14}$$

is the average immediate extrinsic reward expected to be collected by taking action a in state s (see Equation S2). We note that in Equation S14, we used the fact that the goal states are known from the beginning, i.e., $\tilde{G}_i \in \mathcal{S}^{(t)}$ for $i \in \{1,2,3\}$. Hence, the knowledge of the existence of three different goal states with three different rewards has an explicit influence on the MB branch.

To intuitively understand this influence, we first focus on episode 1. Because no transitions to any of the goal states is experienced during episode 1, we have

$$\bar{R}^{(t)}_{\text{ext}}(s,a) = \frac{\epsilon_{\text{known}}(1 + r_1^* + r_2^*)}{\epsilon_{\text{new}} + \epsilon_{\text{known}}|\mathcal{S}^{(t)}| + \tilde{C}^{(t)}_{s,a}}. \tag{S15}$$

$\bar{R}^{(t)}_{\text{ext}}(s,a)$ is closely linked to (approximately) Bayes-optimal exploration bonuses in the RL theory [13] and has two important properties. First, $\bar{R}^{(t)}_{\text{ext}}(s,a)$ is an increasing function of ϵ_{known}. This implies that the expected reward of a transition during episode 1 increases by increasing the prior probability of transition to states in $\mathcal{S}^{(t)}$. This is a direct consequence of our Bayesian approach to estimating the world-model. Second, $\bar{R}^{(t)}_{\text{ext}}(s,a)$ is a decreasing function of $\tilde{C}^{(t)}_{s,a}$. This implies that the expected reward of a state-action pair decreases by experience. Importantly, $\bar{R}^{(t)}_{\text{ext}}(s,a)$ converges to 0 as $\tilde{C}^{(t)}_{s,a} \to \infty$, which makes a link between exploration driven by the MB optimistic initialization and exploration driven by information gain (see Section 4.3).

During episodes 2-5, the exact theoretical analysis of the MB optimistic initialization is rather complex. However, using a few approximation steps for episode 2, we can find a condition for whether the MB extrinsic Q-values show a preference for exploring or leaving the stochastic part (Section 4.5). The condition involves a comparison between the discounted reward value of the discovered goal state $\gamma^2_{\text{ext}} r_{G^*}$ and an optimistic estimate of a reward-to-be-found in the stochastic part $R^{(t)}_{\text{Stoch.}}$. The estimate $R^{(t)}_{\text{Stoch.}}$ depends on r_1^*, r_2^*, γ_{ext}, ϵ_{known}, $|\mathcal{S}^{(t)}|$, and the average leaky count $\bar{C}^{(t)}$ of state-action pairs in the stochastic part (Section 4.5). We show that if $r_{G^*} < r_2^*$, then increasing r_2^* would eventually result in a preference for staying in the stochastic part: If the reward value of a goal state is much greater than the value of the discovered goal state, then the agent prefers to keep exploring the stochastic part. However, for any value of r_2^* and r_{G^*}, increasing $\bar{C}^{(t)}$ would eventually result in a preference for leaving the stochastic part and going towards the already discovered goal: Agents will eventually give up exploration after a sufficiently long and unsuccessful exploration phase. This is another qualitative link between exploration based on the MB optimistic initialization and exploration driven by information gain (see Section 4.3).

Model-free optimistic initialization. Unlike the MB branch, the MF branch does not explicitly know about the existence of different goal states and their values. However, the initial value $Q^{(0)}_{\text{MF,ext}}$ of the MF extrinsic Q-values quantifies an expectation of the reward values in the environment before any interaction with the environment.

During episode 1, no extrinsic reward is received by the agent; hence, for a small enough learning rate

ρ and an optimistic initialization $Q^{(0)}_{\text{MF,ext}} > 0$, the extrinsic reward prediction errors are always negative (Equation S3). As a result, $Q^{(t)}_{\text{MF,ext}}(s,a)$ decreases as an agent keeps taking action a in state s, which motivates the agent to try new actions. This is a well-known mechanism for directed exploration in the machine learning community [6]. Similar to the MB optimistic initialization, the effect of the MF optimistic initialization fades out over time—which makes them both similar to exploration driven by information gain (see Section 4.3).

During episodes 2-5, the exact theoretical analysis of the MF optimistic initialization is complex and dependent on an agent's exact trajectory (because of the eligibility traces). However, whether the MF extrinsic Q-values show a preference for exploring or leaving the stochastic part essentially depends on the reward value of the discovered goal state r_{G^*} and the initialization value $Q^{(0)}_{\text{MF,ext}}$.

To gain some insights, we consider an example: If an agent, starting at $s1$, takes the perfect trajectory of $s1 \to s2 \to s3 \to s4 \to s5 \to s6 \to G^*$ in episode 1, then, given a unit decay factor of the eligibility traces (i.e., $\lambda_{\text{ext}} = 1$), it is easy to see that, in the 1st visit of state 4 in episode 2, the agent prefers the stochastic/bad action over the progressing action if $r_{G^*} < \frac{1}{\gamma^2_{\text{ext}}}(1-\gamma_{\text{ext}})(1+\gamma_{\text{ext}}+\gamma^2_{\text{ext}})Q^{(0)}_{\text{MF,ext}}$. This implies that, even though the MF branch is not explicitly aware of different goal states and their reward values, it can still describe a type of reward optimism through the initialization of Q-values.

2.3 The four algorithms compared in the main text

We considered four hypotheses for how humans explore the environment to search for the goal state, including the most representative exploration strategies in RL [14–16]: (i) seeking novelty, (ii) seeking information gain, (iii) seeking surprise, and (iv) exploration with no intrinsic reward. We formalized the four hypotheses in our framework by using different types of the intrinsic reward function $R_{\text{int},t}$ that maps a transition $(s,a) \to s'$ to an intrinsic reward value $r_{\text{int},t+1} = R_{\text{int},t}(s_t, a_t \to s_{t+1})$.

2.3.1 Novelty-seeking

For an agent seeking novelty (red in Figure 4), we defined the intrinsic reward function as

$$R_{\text{int},t}(s, a \to s') = -\log p^{(t)}_f(s'), \tag{S16}$$

where $p^{(t)}_f(s') = \frac{1+\tilde{C}^{(t)}_{s'}}{1+|\mathcal{S}^{(t)}|+\sum_{s''}\tilde{C}^{(t)}_{s''}}$ is the state frequency with $\tilde{C}^{(t)}_{s'}$ the leaky count of encounters of state s' up to time t (similar to Equation S6): $\tilde{C}^{(t+1)}_{s'} = \kappa\tilde{C}^{(t)}_{s'} + \delta_{s',s_{t+1}}$ with $\tilde{C}^{(0)}_{s'} = 0$; the empirical frequency $p^{(t)}_f$ is denoted by $\tilde{p}_t$ in the main text, for the sake of simplifying the notation.

With this definition, which generalizes earlier works [2] to the case where the number of states is unknown, the least novel states are those that have been encountered most often (i.e., with the highest $\tilde{C}^{(t)}_{s'}$). Moreover, novelty is at its highest value for the unobserved states as we have $\tilde{C}^{(t)}_{s'} = 0$ for any unobserved state $s' \notin \mathcal{S}^{(t)}$. Similar intrinsic rewards have been used in machine learning [17, 18].

To dissociate the effect of exploration by novelty-seeking from optimistic initialization in *episode 1*, we considered $\beta^{(1)}_{\text{MF,ext}} = \beta^{(1)}_{\text{MB,ext}} = 0$ and $Q^{(0)}_{\text{MF,ext}} = 0$; see Section 4.6 for further discussion. Moreover, we put $T_{PS,\text{ext}} = T_{PS,\text{int}} = 100$ (i.e., almost twice the total number of states) to decrease the number of parameters, based on the results of [2] showing the negligible importance of fitting this parameter. Hence, the novelty-seeking algorithm had a total of **27 parameters** (11 main parameters + 14 inverse temperature parameters + 2 biases).

2.3.2 Information-gain-seeking

For an agent seeking information gain (green in Figure 4), we defined the intrinsic reward function as

$$R_{\text{int},t}(s, a \to s') = \mathrm{D}_{\mathrm{KL}}\Big[p^{(t)}(.|s, a)||p^{(t+1)}(.|s, a)\Big], \tag{S17}$$

where D_{KL} is the Kullback-Leibler divergence [19], and $p^{(t+1)}$ is the updated world-model upon observing $(s, a) \to s'$. The dots in Equation S17 denote the dummy variable over which we integrate to evaluate the Kullback-Leibler divergence. Note that if $s' \notin \mathcal{S}^{(t)}$, then there are some technical problems in the naive computation of D_{KL}—since $p^{(t)}$ and $p^{(t+1)}$ have different supports. We dealt with these problems using a more fundamental definition of D_{KL} using the Radon–Nikodym derivative; see Section 4.3 for derivations and see [20] for an alternative heuristic solution. Note that the information gain in Equation S17 has also been interpreted as a measure of surprise (called 'Postdictive surprise' [21]), but it has a behavior radically different from that of the Shannon surprise introduced below for our surprise-seeking agents (Equation S18)—see [22] for an elaborate treatment of the topic. Importantly, the expected (integrated over s') information gain corresponding to a state-action pair (s, a) converges to 0 as $\tilde{C}^{(t)}_{s,a} \to \infty$ (see Section 4.3 for the proof). Similar intrinsic rewards have been used in machine learning [20, 23–25].

Similarly to the case of novelty-seeking, we considered $\beta^{(1)}_{\text{MF,ext}} = \beta^{(1)}_{\text{MB,ext}} = 0$, $Q^{(0)}_{\text{MF,ext}} = 0$, and $T_{PS,\text{ext}} = T_{PS,\text{int}} = 100$; see Section 4.6. Hence, the algorithm seeking information gain also had a total of **27 parameters** (11 main parameters + 14 inverse temperature parameters + 2 biases).

2.3.3 Surprise-seeking

For an agent seeking surprise (orange in Figure 4), we defined the intrinsic reward function as the Shannon surprise (a.k.a. surprisal) [22]

$$R_{\text{int},t}(s, a \to s') = -\log p^{(t)}(s'|s, a), \tag{S18}$$

where $p^{(t)}(s'|s, a)$ is defined in Equation S7. With this definition, the expected (integrated over s') intrinsic reward of taking action a at state s is equal to the entropy of the distribution $p^{(t)}(s'|s, a)$ [19]. If $\epsilon_{\text{new}} < \epsilon_{\text{known}}$, then the most surprising transitions are the ones to unobserved states. Similar intrinsic rewards have been used in machine learning [26, 27].

Similarly to the case of novelty-seeking, we considered $\beta^{(1)}_{\text{MF,ext}} = \beta^{(1)}_{\text{MB,ext}} = 0$, $Q^{(0)}_{\text{MF,ext}} = 0$, and $T_{PS,\text{ext}} = T_{PS,\text{int}} = 100$; see Section 4.6. Hence, the surprise-seeking algorithm had also a total of **27 parameters** (11 main parameters + 14 inverse temperature parameters + 2 biases).

2.3.4 Exploration with no intrinsic reward

As our last alternative algorithm (black in Figure 4), we considered agents with no intrinsic reward:

$$R_{\text{int},t}(s, a \to s') = 0. \tag{S19}$$

Exploratory actions of these agents are purely driven by the MB and MF optimistic initialization described in Section 2.2. As a result, exploration based with no intrinsic reward does not depend on any of the parameters that influence the intrinsically motivated part of the RL algorithm described above, ending up with a total of **19 parameters** (9 main parameters + 8 inverse temperature parameters + 2 biases; considering $T_{PS,\text{ext}} = 100$).

2.4 Model-fitting and model-comparison

To compare different algorithms based on their explanatory power, we did a stratified 3-fold cross-validation [28]: We grouped our 57 human participants into 3 disjoint sets, where all sets had almost

the same number of participants from different reward groups (i.e., 2 CHF, 3 CHF, 4 CHF). For each fold $k \in \{1, 2, 3\}$ of cross-validation, one set of participants was considered as testing set $D_k^{(\text{test})}$ and the union of the other two as the training set $D_k^{(\text{train})}$.

Then, for each model $M \in \{\text{novelty}, \text{inf-gain}, \text{surprise}, \text{no int}\}$ and cross-validation fold $k \in \{1, 2, 3\}$, we fitted the model parameters Φ_M by maximizing log-likelihood of the training data given parameters:

$$\hat{\Phi}_{k,M} = \arg\max_{\Phi_M} \log P(D_k^{(\text{train})} | \Phi_M, M) \tag{S20}$$

where $P(D_k^{(\text{train})} | \Phi_M, M)$ is the probability that $D_k^{(\text{train})}$ is generated by simulating model M with Φ_M (see Equation S12), and $\hat{\Phi}_{k,M}$ is the set of estimated parameters that maximizes that probability. For optimization, we first applied a gradient-free algorithm (Subplex [29]) to perform a broad search of the parameter space, starting from three different initial conditions. The results of this broad search were then used as initialization for a gradient-based algorithm (L-BFGS [30]), which we employed for fine-tuning. To accelerate the initial broad search, we introduced two constraints: (i) setting $b(\text{middle}) = b(\text{right}) = 0$, and (ii) treating $\beta_{\text{MB,ext}}^{(2,r)}$ and $\beta_{\text{MF,ext}}^{(2,r)}$ as independent of the discovered reward r. These constraints were removed in the fine-tuning step. In both steps, we followed standard model-fitting practices (e.g., [31]) and carried out unconstrained optimization by reparameterizing bounded parameters with a logit transformation and positive parameters with an inverse-softplus transformation. To prevent parameter divergence for less sensitive parameters, we additionally included a negligible ℓ_2 regularization term in the loss function (weight 10^{-3}).

We then evaluated all models on the testing set: For each participant n in the testing set $D_k^{(\text{test})}$ of fold k, we evaluated the cross-validated log-likelihood as

$$\hat{\ell}_{n,M} = \log P(D_{k(n)}^{(\text{test})} | \hat{\Phi}_{k,M}, M), \tag{S21}$$

where $D_{k(n)}^{(\text{test})}$ is the data of participant n (which we assumed to be in the testing set of fold k). We then used the cross-validated log-likelihoods in the Bayesian model selection method of [32] with the random effects assumption: We assumed that, with an unknown probability P_M, the data of each participant n was generated by simulating model $M_n = M$. The goal of the model comparison is to infer the probability P_M for all models; the one with the highest P_M is the most probable model for most participants. To do so, we performed Markov Chain Monte Carlo sampling (using Metropolis-Hastening algorithm [33] with 100 chains of length $10'000$) and estimated the joint posterior distribution over P_{novelty}, $P_{\text{inf-gain}}$, P_{surprise}, and $P_{\text{no int}}$. Figure 4B shows the expected value of P_M (the expected posterior probability; Figure 4B1) and the probability of P_M being higher than $P_{M'}$ for all $M' \neq M$ (the protected exceedance probabilities; Figure 4B2). Figure 4D shows the protected exceedance probabilities when the posterior distribution is evaluated conditioned on participants' data in only one of the reward groups. See [2, 34] for a similar approach and [35, 36] for tutorials on the topic.

Finally, for each participant n in the testing set $D_k^{(\text{test})}$ of fold k, we evaluated the accuracy rate of novelty-seeking (Figure 4C) in predicting the participant's actions (conditioned on the past actions) in each episode, i.e., we evaluated the ratio of actions where novelty-seeking with parameter $\hat{\Phi}_{k,\text{novelty}}$ assigned the highest probability to the participant's chosen action; whenever the maximum probability was shared between 2 or 3 actions, we considered the prediction 1/2 or 1/3 correct, respectively (i.e., a random model would have a 33% accuracy rate).

2.5 Posterior predictive checks and model-recovery

For each model $M \in \{\text{novelty}, \text{inf-gain}, \text{surprise}, \text{no int}\}$, we repeated the following steps 1500 times: 1. We picked, with one-third probability, the fitted parameter $\hat{\Phi}_{k,M}$ of fold $k \in \{1,2,3\}$. 2. We picked, with one-third probability, one of the reward conditions (i.e., 2 CHF, 3 CHF, and 4 CHF). 3. We simulated model M with parameters $\hat{\Phi}_{k,M}$ for 5 episodes in our environment, i.e., we sampled a trajectory D from $P(D|\hat{\Phi}_{k,M}, M)$, with the G^* of the environment corresponding to the reward group picked in step 2. As a result, we ended up with 1500 simulated agents (with randomly sampled parameters) for each algorithm.

Depending on their exploration strategy and parameters, some simulated agents either continued to explore the stochastic part of the environment or repeatedly fell into the trap states. Hence, we stopped simulations of each episode after 3000 actions; note that the median number of actions taken by human participants is less than 100 (Figure 2B-C). Accordingly, we considered the simulated agents who took more than 3000 actions in any of the 5 episodes to be similar to the human participants who quit the experiment and excluded them from further analyses. Moreover, we applied the same criterion that we used for the human participants and excluded, separately for each algorithm, the simulated agents who took more than 3 times the group-average number of actions in episodes 2-5 to finish the experiment. In total, we excluded $13 \pm 1\%$ of agents seeking novelty, $18 \pm 1\%$ of agents seeking information-gain, $35 \pm 1\%$ of agents seeking surprise, and $42 \pm 2\%$ of agents without intrinsic rewards. For reference, we had excluded $10 \pm 4\%$ of human participants. These numbers show that the distribution of the number of actions for all *but* novelty-seeking simulated agents had a heavy tail. Our main conclusions in the main text do not change even if we include the simulations corresponding to this heavy tail (Figure S4).

In our main analyses, we focused on the remaining simulated agents. Figure 2D-F shows the data statistics of simulated novelty-seeking agents compared to human participants; The replication of Figure 2D for all algorithms is shown in Figure 5A. Figure 5B shows the median and the average relative error (absolute difference divided by SE) of different algorithms in reproducing 43 group-level statistics: (1) number of actions in episode 1, (2-5) fractions of trials spent in trap states and stochastic parts during the 1st and 2nd halves of episode 1 (Figure 2A), (6-9) median number of actions in episodes 2-5 for each reward group and its correlation with reward value (Figure 2B1), (10-13) fraction of time-steps spent in the stochastic part in episodes 2-5 for each reward group and its correlation with reward value (Figure 2B2), (14-16) correlation of episode length with episode number for each reward group (e.g., Figure 2C for the 2 CHF group), (17-19) correlation of the fraction of time-steps spent in the stochastic part with the episode number for each reward group, and (20-43) the ratio of taking different actions (2 possibilities, i.e., progressing action and self-looping/stochastic action) in different progressing states (3 possibilities, i.e., states 1-3, state 4, and states 5-6) and in different periods of the experiment (4 possibilities, i.e., episode 1 for all participants and episodes 2-5 separately for each reward group). See Figure S3 for details.

Finally, for the simulated data of each algorithm, we repeated the model selection procedure (i.e., 3-fold cross-validation followed by Bayesian model selection) on the action choices of five groups of 60 simulated agents (20 from each participant group: 2 CHF, 3 CHF, and 4 CHF). To accelerate this model-recovery step, we initialized the optimization with the parameter estimates obtained from the fit to all real participants. We always successfully recovered the model that had generated the data, using almost the same number of simulated agents (60) as human participants (57). See insets in Figure 4B for confusion matrices.

3 Supplementary Results

3.1 Different intrinsic rewards react differently to stochasticity

To confirm that the stochastic part of our experimental paradigm preserves the essential features of a noisy TV [14, 27], we simulated three groups of RL agents (500 agents per group) exploring our environment by seeking (i) surprise, (ii) novelty, or (iii) information gain. As a control group, we also simulated 500 random agents taking each action with an equal probability of 1/3.

We aimed to quantify the isolated effect of the intrinsic reward on the simulated agents' attraction to the stochastic part. To achieve this, we considered the most efficient version of each exploration strategy by employing the model-based branch of our algorithm in Algorithm 1. Additionally, we removed the extrinsic-reward-seeking component of the algorithm; this would allow us to analyze the purely exploratory behavior of the RL agents in isolation (similar to [26, 27]).

The resulting algorithm included a total of 7 parameters: $\{\gamma_{\text{int}}, \kappa, \epsilon_{\text{new}}, \epsilon_{\text{known}}, T_{PS,\text{int}}, \beta^{(1)}_{\text{MB,int}}, \beta^{(2)}_{\text{MB,int}}\}$. To avoid arbitrariness in parameter selection, we assumed perfect model-building by setting $\kappa = 1$ and nearly perfect planning by setting $T_{PS,\text{int}} = 100$. Furthermore, we chose the discount factor γ_{int} and the prior parameters ϵ_{new} and ϵ_{known} based on the range of fitted parameters reported by [2]: $\gamma_{\text{int}} = 0.70$, $\epsilon_{\text{new}} = 10^{-5}$, and $\epsilon_{\text{known}} = 10^{-4}$. Finally, after fixing these parameter values, we fine-tuned $\beta^{(1)}_{\text{MB,int}}$ to minimize the average length of episode 1; in other words, we fine-tuned $\beta^{(1)}_{\text{MB,int}}$ such that the agents would find the goal as fast as possible (Figure S1). As expected, agents seeking information gain were fastest in finding the goal state (Figure S1B1), followed by the novelty-seeking (Figure S1A1) and surprise-seeking agents (Figure S1C1). All intrinsically motivated agents were faster than random agents (Figure S1D1). Importantly, already in episode 1, all intrinsically motivated agents learned to avoid the trap states and were attracted to the stochastic part of the environment (Figure S1A2-C2). The random agents, however, spent most of their time in the trap states and only a marginal fraction in the stochastic part (Figure S1D2).

Given the parameters fine-tuned for episode 1, we then simulated the agents for an additional 4 episodes with $\beta^{(2)}_{\text{MB,int}} = \beta^{(1)}_{\text{MB,int}}$. Each episode ended when agents reached the goal state G^*, even though no extrinsic reward was associated with G^*. Depending on their exploration strategy, some simulated agents continued to explore the stochastic part of the environment and did not escape it. Hence, we stopped simulations of each episode after 3000 actions. We characterized the exploratory behavior of different agents during episodes 2–5 by measuring the search duration (Figure S2A) and the fraction of time-steps spent in the stochastic part (Figure S2B). For agents seeking information gain, both the search duration and the fraction of time-steps in the stochastic part decreased over episodes (Figure S2A3 and B3). Conversely, novelty- and surprise-seeking agents exhibited the opposite pattern (Figure S2A1-A2 and B1-B2). Notably, surprise-seeking agents were often (i.e., in $> 50\%$ of simulations in episode 5) stuck in the stochastic part, failing to escape within 3000 actions (Figure S2A1). By design, random agents exhibited consistent behavior across episodes (Figure S2A4 and B4): They had a persistently higher search duration, compared to novelty- and information-gain-seeking agents (Figure S2A4 versus Figure S2A2-A3), but spent only a marginal fraction of their time in the stochastic part of the environment (Figure S2B4 versus Figure S2B1-B3).

These findings confirm that the stochastic part of our experimental paradigm effectively replicates the distinct exploration patterns previously associated with different intrinsic rewards [14].

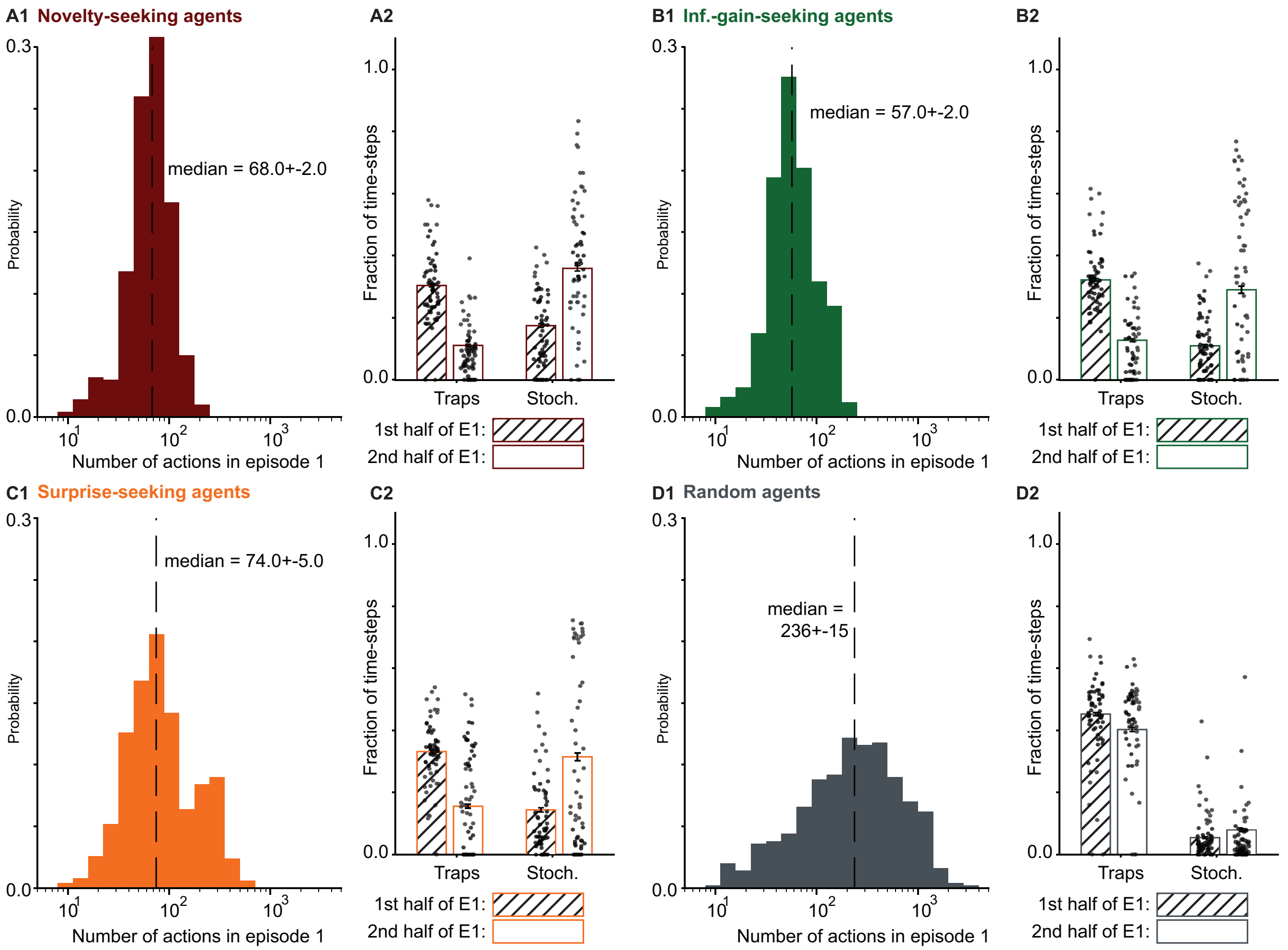

Figure S1: **Efficient exploration driven by different intrinsic rewards rapidly finds the goal state in episode 1 (Supplementary to Figure 1). A1-D1.** Histogram of the number of actions in episode 1 for agents simulated by different algorithms (500 simulations for each algorithm). **A2-D2.** Fraction of time-steps spent in the stochastic part during the 1st and the 2nd half of episode 1 (similar to Figure 2A). Error bars show the SEMean. Single dots show the data of (60 out of 500) individual simulations.

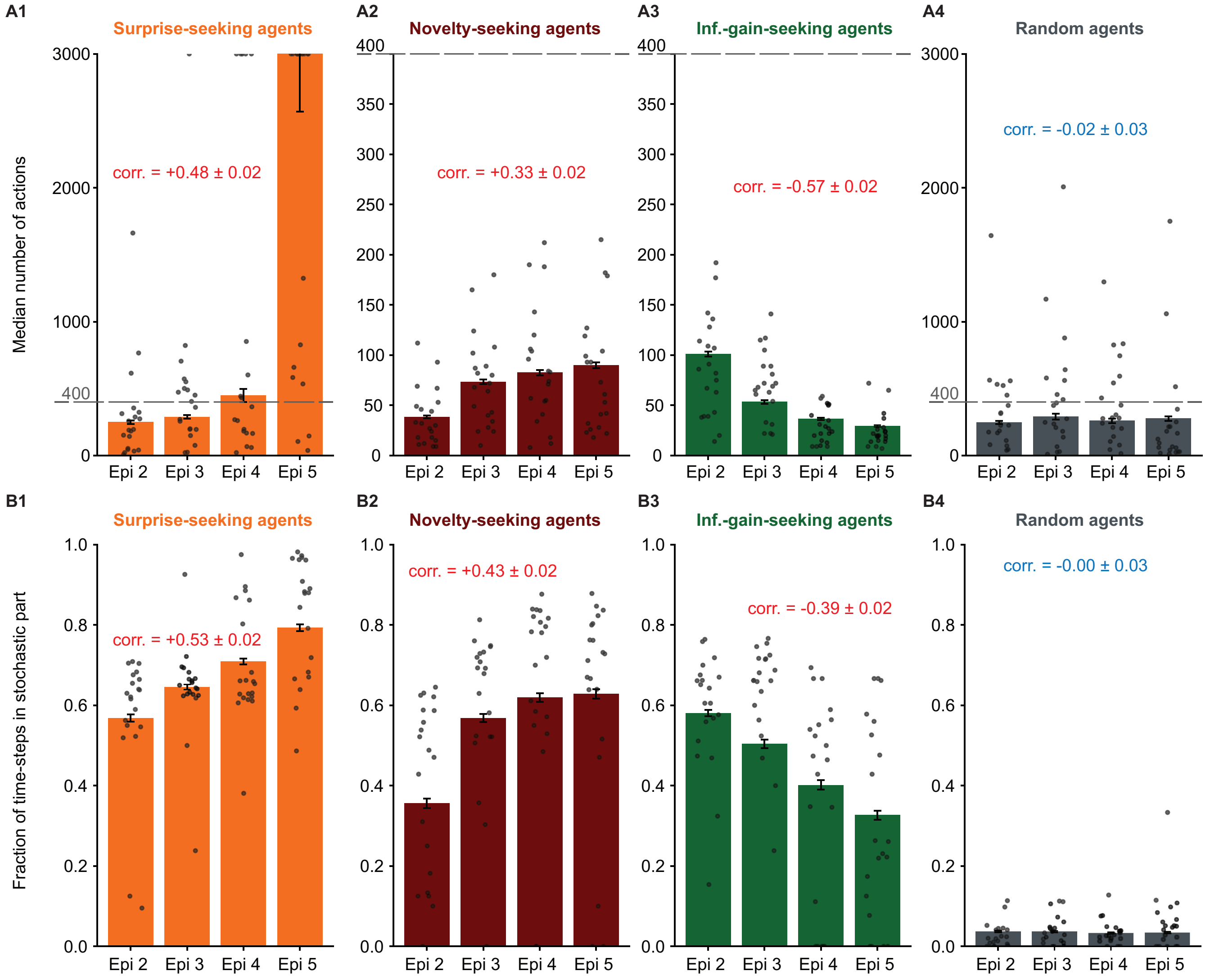

Figure S2: **Efficient exploration driven by different intrinsic rewards shows different patterns of attraction to the stochastic part (Supplementary to Figure 1). A.** Median number of actions in episodes 2-5 for agents simulated by different algorithms (500 simulations for each algorithm). Error bars show the SEMed. Correlations denote the average (across simulations) Pearson correlation between the number of actions and the episode number. **B.** fraction of time-steps spent in the stochastic part in episodes 2-5. Error bars show the SEMean. In all panels, single dots show the data of (20 out of 500) individual simulations. Correlations denote the average (across simulations) Pearson correlation between the fraction of time-steps in the stochastic part and the episode number.

3.2 Detailed summary statistics for posterior predictive checks

For model selection based on Posterior Predictive Checks (PPC), we reported the median relative error of different algorithms in reproducing 43 summary statistics of human data (Figure 5 in the main text). Figure S3 displays the relative error for all summary statistics separately. Novelty-seeking most accurately reproduces the majority of the summary statistics of human action choices (Figure S3; 1st row versus the others). However, as mentioned in the Discussion in the main text, these replications are far from perfect.

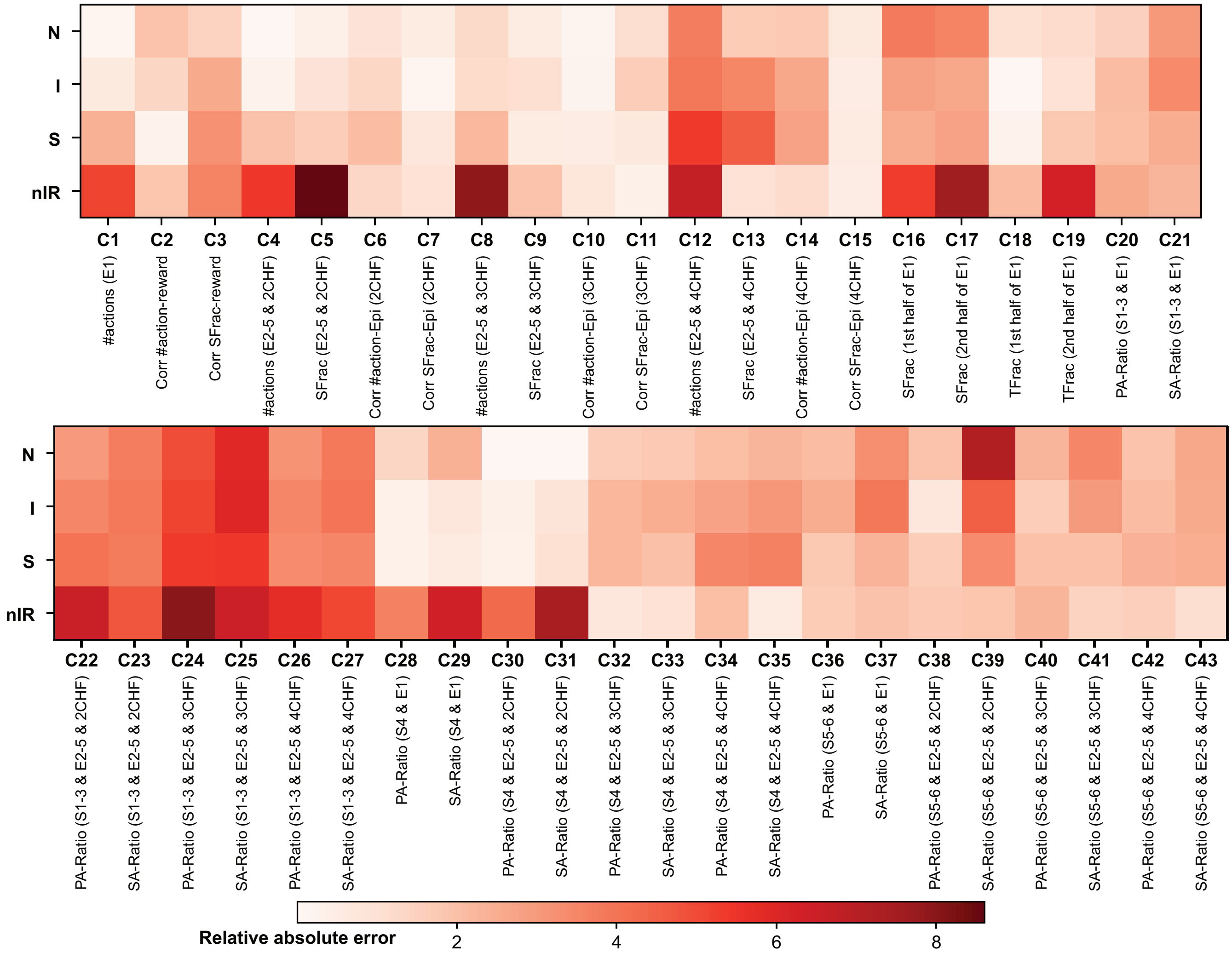

Figure S3: **Relative error of different algorithms in replicating 43 summary statistics of human data (Supplementary to Figure 5).** The heatmap displays the relative error, calculated as the absolute difference in the summary statistics divided by their standard errors. Each row corresponds to one of the four algorithms: novelty-seeking (N), information-gain-seeking (I), surprise-seeking (S), and exploration with no intrinsic reward (nIR). Each column (C1-C43) corresponds to one of the 44 summary statistics: the median number of actions in episode 1 (C1); correlation between the reward value of G^* and the number of actions in episodes 2-5 (C2) or the fraction of time-steps spent in the stochastic states in episodes 2-5 (C3); the median number of actions (C4, C8, and C12) and the average fraction of time in the stochastic states (C5, C9, and C13) during episodes 2-5 for different reward groups (2CHF, 3CHF, and 4CHF), respectively; correlation between the episode number and the number of actions (C6, C10, and C14) or the fraction of time in the stochastic states (C7, C11, and C15) during episodes 2-5 for different reward groups (2CHF, 3CHF, and 4CHF), respectively; fraction of time-steps spent in the stochastic part (C16 and C17) and the trap states (C18 and C19) during the two parts (the 1st and 2nd halves) of episode 1, respectively; the ratio (C20-C43) of taking different actions (2 possibilities, i.e., progressing action and self-looping/stochastic action, abbreviated by PA and SA, respectively) in different progressing states (3 possibilities, i.e., states 1-3, state 4, and states 5-6, abbreviated by S1-3, S4, and S5-6) and in different periods of the experiment (4 possibilities, i.e., episode 1 for all participants, abbreviated by E1, and episodes 2-5 separately for each reward group, indexed by E2-5 & 2CHF, 3CHF, or 4CHF, respectively).

3.3 Posterior predictive checks with no exclusion criterion

Figure S4 is a replication of Figure 5 in the main text but with including all simulations—even those for which the simulation was stopped at 3'000 actions (see Section 2.5).

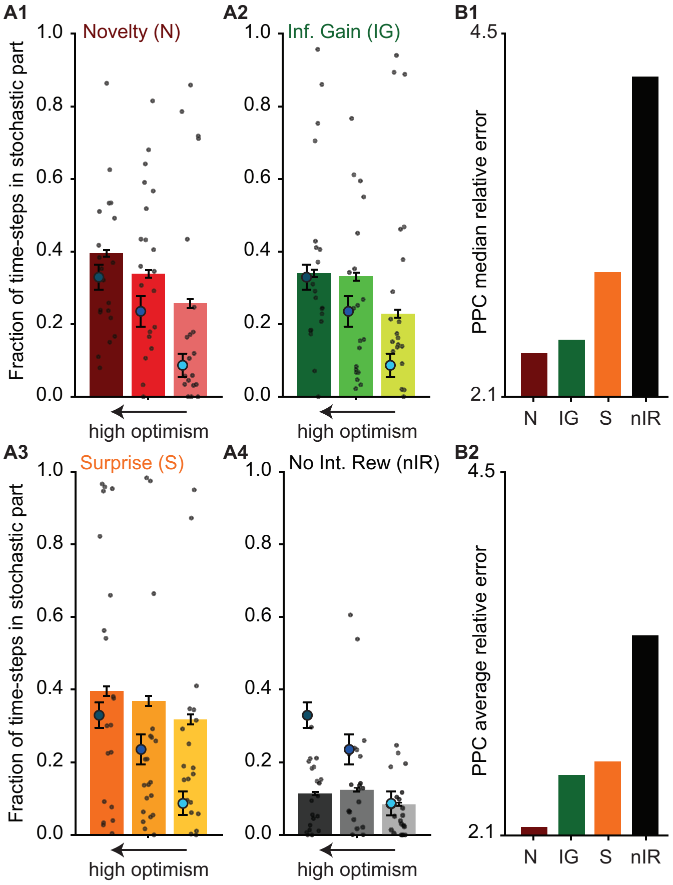

Figure S4: **PPC-based model comparison without excluding outlier simulations (Supplementary to Figure 5).** This figure follows the same structure as Figure 5 in the main text. Including outlier simulations degrades both the qualitative and quantitative performance of all algorithms; however, novelty-seeking agents remain the most similar to human participants.

3.4 Model-selection with uniformly leaking counts

In our main computational model, we accounted for imperfect model-building by incorporating leakiness in the transition counts (Equation S22). Specifically, after taking action a_t in state s_t, we assumed that the agent decreases the counts $\tilde{C}_{s,a,s'}$ corresponding to the state-action pair (s_t, a_t). However, we kept the counts associated with all other state-action pairs unchanged. This modeling choice was motivated by previous findings showing that participants' estimates of transition probabilities in a given state s are not affected by resets in a different state s' [2].

As a control, we also considered an alternative approach inspired by [37, 38], in which all counts leak uniformly, i.e.,

$$\tilde{C}^{(t+1)}_{s,a,s'} = \begin{cases} \kappa \tilde{C}^{(t)}_{s,a,s'} + \delta_{s',s_{t+1}} & \text{if } s = s_t,\ a = a_t\,, \\ {\color{blue}\kappa} \tilde{C}^{(t)}_{s,a,s'} & \text{otherwise,} \end{cases} \tag{S22}$$

where κ in the second line is colored in blue to emphasize the difference between Equation S6 and Equation S22.

To test whether and how the type of leakiness changes our results, we repeated our model selection procedure using Equation S22 in place of Equation S6. To accelerate this step, we initialized the optimization with the fitted parameters from the original model selection. The results of the new model selection confirm our main conclusions in the main text (Figure S5). Additionally, we observed that the uniform leak further impairs the performance of both surprise-seeking and information-gain-seeking algorithms.

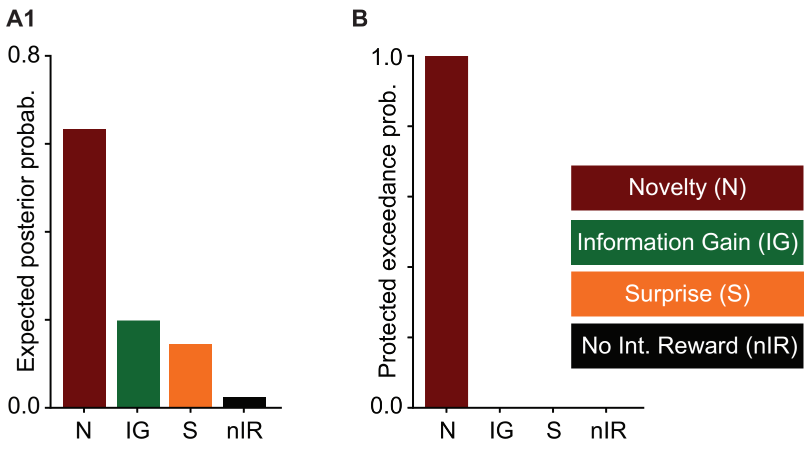

Figure S5: **Model-selection results repeated for the algorithm with uniformly leaking counts (Supplementary to Figure 4B in the main text). A.** The expected posterior probability quantifies the proportion of participants whose behavior is best explained by each algorithm (regarding cross-validated log-likelihoods). **B.** Protected Exceedance Probability quantifies the probability of each model being more frequent than the others among participants.

4 Supplementary Methods: Derivations and theoretical analyses

4.1 Model-building in an environment of unknown size

We used ideas from non-parametric Bayesian inference [1, 39] and Dirichlet processes [40] to derive a Bayesian estimate $p^{(t)}(s'|s,a)$ of the transition probabilities in an environment of unknown size.

4.1.1 Time-dependent base distribution as the expected prior

Consider the 1st time an agent takes action a at state s. Which state s' does the agent expect to visit next, given that it has zero experience for taking action a at state s? There are two possibilities: (i) s' is one of the already known state, i.e., $s' \in \mathcal{S}^{(t)}$, or (ii) s' is one of the infinitely many imaginable states $\mathcal{S}$ that the agent has not observed yet, i.e., $s' \notin \mathcal{S}^{(t)}$. We assume that the agent considers different weights for these two possibilities even in the prior distribution. We give a precise definition of this prior distribution in the following subsection, but we first need to define our *time dependent base distribution* [40], which we will use later.

We define the probability measure H as a continuous probability distribution (i.e., without any atom) on the space of all imaginable states $\mathcal{S}$—e.g., the space of all images that can appear on the computer screen. Our results are independent of the exact shape of H—as long as it is a *continuous* probability distribution. We define the time-dependent base distribution on $\mathcal{S}$ as

$$H^{(t)} := \frac{\epsilon_{\text{new}}}{\epsilon_{\text{new}} + \epsilon_{\text{known}}|\mathcal{S}^{(t)}|} H + \frac{\epsilon_{\text{known}}}{\epsilon_{\text{new}} + \epsilon_{\text{known}}|\mathcal{S}^{(t)}|} \sum_{s \in \mathcal{S}^{(t)}} \delta_s, \tag{S23}$$

where δ_s is the Dirac measure at s, ϵ_{known} and ϵ_{new} are the weights for combining the two possibilities of (i) transition to a known state $s' \in \mathcal{S}^{(t)}$ and (ii) transition to a new and unknown state $s' \in \mathcal{S}$, respectively. In the next section, we use this base distribution as $p^{(t)}(.|s,a)$ for any state-action pair (s,a) that has not been experienced before.

4.1.2 Derivation of the world-model

We indicate the matrix of transition probabilities as a parameter Θ that fully summarizes the environment. Then, given underlying $\Theta = \theta : \mathcal{S} \times \mathcal{A} \to \text{Measures}[\mathcal{S}]$, we have

$$\mathbb{P}(S_{t+1} = s'|S_t = s, A_t = a, \Theta = \theta) := \theta_{s,a}(s') \tag{S24}$$

for any s and $s' \in \mathcal{S}$ and $a \in \mathcal{A}$. Given the sequence of states $S_{1:t} = s_{1:t}$ and actions $A_{1:t-1} = a_{1:t-1}$, an agent's belief about the transition matrix θ is defined as the posterior

$$q^{(t)}(\theta) := \mathbb{P}^{(t)}(\theta|s_{1:t}, a_{1:t-1}) \propto \mathbb{P}^{(t)}(\theta)\mathbb{P}^{(t)}(s_{2:t}|\theta, a_{1:t-1}, s_1) = \mathbb{P}^{(t)}(\theta) \prod_{t'=1}^{t-1} \theta_{s_{t'},a_{t'}}(s_{t'+1}), \tag{S25}$$

where the prior $\mathbb{P}^{(t)}$ is a time-dependent prior distribution over transition probabilities. We assume that $\Theta_{s,a}$s are *a priori* i.i.d. samples of a Dirichlet process prior [40] with the base distribution $H^{(t)}$ and a time-dependent concentration parameter $\alpha^{(t)}$, that is, for any finite and countable $\mathcal{S}' \subseteq \mathcal{S}$,

$$\mathbb{P}^{(t)}\big(\{\theta_{s,a} : s \in \mathcal{S}', a \in \mathcal{A}\}\big) = \prod_{s \in \mathcal{S}', a \in \mathcal{A}} \text{DP}(\theta_{s,a}; \alpha^{(t)}, H^{(t)}), \tag{S26}$$

where DP stands for Dirichlet Process. $H^{(t)}$ is the prior expected value of Θ and can be seen as a prior estimate of transition probabilities, and $\alpha^{(t)}$ denotes how many samples this estimate is worth [33, 40].

Putting a weight of ϵ_{known} for each known state and ϵ_{new} for all unknown states (Equation S23), we end up with $\alpha^{(t)} = \epsilon_{\text{new}} + \epsilon_{\text{known}}|\mathcal{S}^{(t)}|$ as the number of samples that $H^{(t)}$ is worth.

It is straightforward to show that the posterior distribution $q^{(t)}$ has the same form as the prior [40], that is, for any finite and countable $\mathcal{S}' \subseteq \mathcal{S}$,

$$q^{(t)}\big(\{\theta_{s,a} : s \in \mathcal{S}', a \in \mathcal{A}\}\big) = \prod_{s\in\mathcal{S}', a\in\mathcal{A}} \mathrm{DP}\Big(\theta_{s,a}\,;\, \alpha^{(t)} + C^{(t)}_{s,a}\,,\, \frac{\alpha^{(t)}}{\alpha^{(t)} + C^{(t)}_{s,a}} H^{(t)} + \frac{1}{\alpha^{(t)} + C^{(t)}_{s,a}} \sum_{s'\in\mathcal{S}^{(t)}} C^{(t)}_{s,a,s'}\delta_{s'}\Big), \tag{S27}$$

where $C^{(t)}_{s,a}$ is the number of times action a has been taken at state s' until time t, and $C^{(t)}_{s,a,s'}$ is the number of times transition $(s,a) \to s'$ has been experienced. We consider the posterior expected value of Θ as an agent's estimate of the world-model [40]:

$$\begin{aligned} p^{(t)}(s'|s,a) := \hat{\theta}^{(t)}_{s,a}(s') := \mathbb{E}_{q^{(t)}}[\Theta_{s,a}(s')] &= \frac{\alpha^{(t)}}{\alpha^{(t)} + C^{(t)}_{s,a}} H^{(t)}(s') + \frac{1}{\alpha^{(t)} + C^{(t)}_{s,a}} \sum_{s''\in\mathcal{S}^{(t)}} C^{(t)}_{s,a,s''}\delta_{s''}(s') \\ &= \frac{\alpha^{(t)}(1-c_t)}{\alpha^{(t)} + C^{(t)}_{s,a}} H(s') + \frac{1}{\alpha^{(t)} + C^{(t)}_{s,a}} \sum_{s''\in\mathcal{S}^{(t)}} \Big(\frac{\alpha^{(t)}c_t}{|\mathcal{S}^{(t)}|} + C^{(t)}_{s,a,s''}\Big)\delta_{s''}(s'), \end{aligned} \tag{S28}$$

where we used Equation S23 and defined $c_t := \frac{\epsilon_{\text{known}}|\mathcal{S}^{(t)}|}{\epsilon_{\text{new}} + \epsilon_{\text{known}}|\mathcal{S}^{(t)}|}$ to shorten the notation. Equation S28 can be simplified and written as

$$p^{(t)}(s'|s,a) = \hat{\theta}^{(t)}_{s,a}(s') = \begin{cases} \frac{\epsilon_{\text{known}} + C^{(t)}_{s,a,s'}}{\epsilon_{\text{new}} + \epsilon_{\text{known}}|\mathcal{S}^{(t)}| + C^{(t)}_{s,a}} & \text{if} \quad s' \in \mathcal{S}^{(t)}\,, \\ \frac{\epsilon_{\text{new}}}{\epsilon_{\text{new}} + \epsilon_{\text{known}}|\mathcal{S}^{(t)}| + C^{(t)}_{s,a}} & \text{if} \quad s' = s_{\text{new}}\,. \end{cases} \tag{S29}$$

where by $s' = s_{\text{new}}$ we mean $s' \notin \mathcal{S}^{(t)}$, i.e.,

$$\hat{\theta}^{(t)}_{s,a}(s_{\text{new}}) := \mathbb{E}_{\Theta_{s,a}\sim q^{(t)}}\Big[\mathbb{E}_{S'\sim\Theta_{s,a}}[\mathcal{I}_{S'\notin\mathcal{S}^{(t)}}]\Big] = \frac{\alpha^{(t)}(1-c_t)}{\alpha^{(t)} + C^{(t)}_{s,a}} \int_{s'\notin\mathcal{S}^{(t)}} H(s')ds' = \frac{\alpha^{(t)}(1-c_t)}{\alpha^{(t)} + C^{(t)}_{s,a}}. \tag{S30}$$

For the case of $\epsilon_{\text{new}} = 0$, $\epsilon_{\text{known}} = \epsilon$, and $\mathcal{S}^{(t)} = \mathcal{S}$ being a finite and countable set, the transition matrix is the same as the transition matrix conventionally used for finite state-spaces [2, 13]. For the case of $\epsilon_{\text{known}} = 0$, the transition matrix has the form of a Chinese restaurant process [40, 41].

To account for imperfect model-building, we use leaky counts $\tilde{C}^{(t)}_{s,a,s'}$ and $\tilde{C}^{(t)}_{s,a} = \sum_{s'} \tilde{C}^{(t)}_{s,a,s'}$ instead of $C^{(t)}_{s,a}$ and $C^{(t)}_{s,a,s'}$, where $\tilde{C}^{(t)}_{s,a,s'}$ is recursively updated via

$$\tilde{C}^{(t+1)}_{s,a,s'} = \begin{cases} \kappa\tilde{C}^{(t)}_{s,a,s'} + \delta_{s',s_{t+1}} & \text{if}\, s = s_t\,,\ a = a_t \\ \tilde{C}^{(t)}_{s,a,s'} & \text{otherwise}, \end{cases} \tag{S31}$$

where δ is the Kronecker delta function, $\tilde{C}^{(0)}_{s,a,s'} = 0$, and $\kappa \in [0,1]$ is the leak parameter; this is a common modeling choice in neuroscience and psychology [8, 9, 42]; see Section 3.4 for an alternative approach. If $\kappa = 1$, then $\tilde{C}^{(t+1)}_{s,a,s'} = C^{(t+1)}_{s,a,s'}$.

One may argue that the whole Bayesian formulation could be avoided by considering Equation S29 as the starting point—similar to how we present the model Section 2.1. However, as we will see in the next two sections, Equation S29 without the Bayesian formulation of this section is not enough for (i) deriving the update rule for the model-based branch in Section 4.2 or (ii) evaluating information gain in Section 4.3.

4.2 Prioritized sweeping for updating the MB Q-values

Given a reward function R (i.e., R_{ext} for the extrinsic reward or $R_{\text{int},t}$ for the intrinsic reward) the Bellman equations [6, 43] are

$$Q^{(t)}(s,a) = \mathbb{E}_{S' \sim \hat{\theta}^{(t)}_{s,a}} \Big[R_{s,a}(S') + \gamma \underbrace{\max_{a' \in \mathcal{A}} Q^{(t)}(S', a')}_{=: V^{(t)}(s')} \Big], \tag{S32}$$

where $Q^{(t)}$ is the Q-value (i.e., $Q^{(t)}_{\text{MB,ext}}$ for the extrinsic reward or $Q^{(t)}_{\text{MB,int}}$ for the intrinsic reward), $\gamma \in [0,1)$ is the discount factor (i.e., γ_{ext} for the extrinsic reward or γ_{int} for the intrinsic reward), and we denote $R(s, a \to s')$ by $R_{s,a}(s')$ to shorten the notation.

We assume that $R_{s,a}(s')$ is the same for all $s' \notin \mathcal{S}^{(t)}$. Additionally, we note that $\hat{\theta}^{(t)}_{s,a}(s')$ is also the same for all $s' \notin \mathcal{S}^{(t)}$ or $s \notin \mathcal{S}^{(t)}$ (Equation S29). Hence, $Q^{(t)}(s,a)$ takes the same value for all $s \notin \mathcal{S}^{(t)}$ and for all actions $a \in \mathcal{A}$; we denote this value by $V^{(t)}(s_{\text{new}}) = Q^{(t)}(s_{\text{new}}, a)$.

Accordingly, Equation S32 can be re-written as

$$Q^{(t)}(s,a) = \sum_{s' \in \mathcal{S}^{(t)}} \hat{\theta}^{(t)}_{s,a}(s') \Big(R_{s,a}(s') + \gamma V^{(t)}(s') \Big) + \hat{\theta}^{(t)}_{s,a}(s_{\text{new}}) \Big(R_{s,a}(s_{\text{new}}) + \gamma V^{(t)}(s_{\text{new}}) \Big). \tag{S33}$$

Noting that $V^{(t)}(s_{\text{new}}) = Q^{(t)}(s_{\text{new}}, a)$ and $R_{s_{\text{new}}}(s') := R_{s_{\text{new}},a}(s')$ are independent of a, we can find $V^{(t)}(s_{\text{new}})$ by solving

$$\begin{aligned} V^{(t)}(s_{\text{new}}) &= \sum_{s' \in \mathcal{S}^{(t)}} \hat{\theta}^{(t)}_{s_{\text{new}}}(s') \Big(R_{s_{\text{new}}}(s') + \gamma V^{(t)}(s') \Big) + \hat{\theta}^{(t)}_{s_{\text{new}}}(s_{\text{new}}) \Big(R_{s_{\text{new}}}(s_{\text{new}}) + \gamma V^{(t)}(s_{\text{new}}) \Big) \\ &= \frac{\epsilon_{\text{known}}}{\epsilon_{\text{new}} + \epsilon_{\text{known}} |\mathcal{S}^{(t)}|} \sum_{s' \in \mathcal{S}^{(t)}} \Big(R_{s_{\text{new}}}(s') + \gamma V^{(t)}(s') \Big) + \frac{\epsilon_{\text{new}}}{\epsilon_{\text{new}} + \epsilon_{\text{known}} |\mathcal{S}^{(t)}|} \Big(R_{s_{\text{new}}}(s_{\text{new}}) + \gamma V^{(t)}(s_{\text{new}}) \Big). \end{aligned} \tag{S34}$$

The solution to Equation S34 is given by

$$\begin{aligned} V^{(t)}(s_{\text{new}}) &= \frac{\epsilon_{\text{known}}}{(1-\gamma)\epsilon_{\text{new}} + \epsilon_{\text{known}} |\mathcal{S}^{(t)}|} \sum_{s' \in \mathcal{S}^{(t)}} \Big(R_{s_{\text{new}}}(s') + \gamma V^{(t)}(s') \Big) + \frac{\epsilon_{\text{new}}}{(1-\gamma)\epsilon_{\text{new}} + \epsilon_{\text{known}} |\mathcal{S}^{(t)}|} R_{s_{\text{new}}}(s_{\text{new}}) \\ &= W^{(t)}_{\text{known}} \sum_{s' \in \mathcal{S}^{(t)}} \Big(R_{s_{\text{new}}}(s') + \gamma V^{(t)}(s') \Big) + W^{(t)}_{\text{new}} R_{s_{\text{new}}}(s_{\text{new}}), \end{aligned} \tag{S35}$$

where in the last line we shortened the notation by defining constants

$$W^{(t)}_{\text{known}} := \frac{\epsilon_{\text{known}}}{(1-\gamma)\epsilon_{\text{new}} + \epsilon_{\text{known}} |\mathcal{S}^{(t)}|} \quad \text{and} \quad W^{(t)}_{\text{new}} := \frac{\epsilon_{\text{new}}}{(1-\gamma)\epsilon_{\text{new}} + \epsilon_{\text{known}} |\mathcal{S}^{(t)}|}. \tag{S36}$$

Finally, we combine Equation S33 and Equation S35 to derive a set of equations for the Q-values that depend *only* on states in $\mathcal{S}^{(t)}$:

$$\begin{aligned} Q^{(t)}(s,a) =& \gamma \sum_{s' \in \mathcal{S}^{(t)}} \Big(\hat{\theta}^{(t)}_{s,a}(s') + \gamma \hat{\theta}^{(t)}_{s,a}(s_{\text{new}}) W^{(t)}_{\text{known}} \Big) V^{(t)}(s') + \sum_{s' \in \mathcal{S}^{(t)}} \hat{\theta}^{(t)}_{s,a}(s') R_{s,a}(s') + \\ & \hat{\theta}^{(t)}_{s,a}(s_{\text{new}}) \Big(R_{s,a}(s_{\text{new}}) + \gamma W^{(t)}_{\text{new}} R_{s_{\text{new}}}(s_{\text{new}}) + \gamma W^{(t)}_{\text{known}} \sum_{s' \in \mathcal{S}^{(t)}} R_{s_{\text{new}}}(s') \Big). \end{aligned} \tag{S37}$$

To approximate the solution to this set of equations, we use prioritized sweeping [6, 10, 44]—primarily for practical reasons and implementation efficiency. The modified algorithm is presented in Alg. 4 in which

we use the QUpdate operator defined as

$$\begin{aligned}
&Q\text{Update}(s, a; \gamma, \hat{\theta}, V, R, W_{\text{known}}, W_{\text{new}}, \tilde{\mathcal{S}}) \\
&\quad := \gamma \sum_{s' \in \tilde{\mathcal{S}}} \Big(\hat{\theta}_{s,a}(s') + \gamma \hat{\theta}_{s,a}(s_{\text{new}}) W_{\text{known}} \Big) V(s') + \sum_{s' \in \tilde{\mathcal{S}}} \hat{\theta}_{s,a}(s') R_{s,a}(s') + \\
&\quad \hat{\theta}_{s,a}(s_{\text{new}}) \Big(R_{s,a}(s_{\text{new}}) + \gamma W_{\text{new}} R_{s_{\text{new}}}(s_{\text{new}}) + \gamma W_{\text{known}} \sum_{s' \in \tilde{\mathcal{S}}} R_{s_{\text{new}}}(s') \Big).
\end{aligned} \tag{S38}$$

4.3 Derivation of information gain

Information-gain-seeking algorithms [20, 24, 45] consider the intrinsic reward as the amount of change in the world-model $\hat{\theta}^{(t)}_{s,a}$ upon observing the transition $(s, a) \to s'$, often defined as

$$R_{\text{int},t}(s, a \to s') = IG^{(t)}(s, a \to s') = \text{D}_{\text{KL}}\Big[\ \hat{\theta}^{(t)}_{s,a} \ ||\ \hat{\theta}^{(t+1)}_{s,a\to s'}\ \Big], \tag{S39}$$

where $\hat{\theta}^{(t+1)}_{s,a\to s'}$ is $\hat{\theta}^{(t+1)}_{s,a}$ if $S_{t+1} = s'$, and D_{KL} is the Kullback-Leibler divergence [19]. In different contexts, $IG^{(t)}(s, a \to s')$ is also called Postdictive surprise [21], but it has a fundamentally different behavior from the *prediction* surprise $-\log \hat{\theta}^{(t)}_{s,a}(s')$ that we used for our surprise-seeking algorithm (see [22] for more discussion).

If $s' \notin \mathcal{S}_t$, the naïve definition of D_{KL} cannot be used in Equation S39 because $\hat{\theta}^{(t)}_{s,a}$ and $\hat{\theta}^{(t+1)}_{s,a\to s'}$ has different supports for their atoms. To resolve this issue, [20] propose a padding mechanism as a heuristic solution. We use a more general definitions of D_{KL} as the expected Radon–Nikodym derivative of $\hat{\theta}^{(t)}_{s,a}$ with respect to $\hat{\theta}^{(t+1)}_{s,a\to s'}$ that is well-defined in our Bayesian framework:

$$\text{D}_{\text{KL}}\Big[\ \hat{\theta}^{(t)}_{s,a} \ ||\ \hat{\theta}^{(t+1)}_{s,a\to s'}\ \Big] = \mathbb{E}_{S'' \sim \hat{\theta}^{(t)}_{s,a}} \left[\frac{d\hat{\theta}^{(t)}_{s,a}}{d\hat{\theta}^{(t+1)}_{s,a\to s'}}(S'') \right], \tag{S40}$$

where $\frac{d\hat{\theta}^{(t)}_{s,a}}{d\hat{\theta}^{(t+1)}_{s,a\to s'}}(S'')$ is the Radon–Nikodym derivative of $\hat{\theta}^{(t)}_{s,a}$ with respect to $\hat{\theta}^{(t+1)}_{s,a\to s'}$ at S''; we note that $\hat{\theta}^{(t)}_{s,a}$ is always absolutely continuous with respect to $\hat{\theta}^{(t+1)}_{s,a\to s'}$, which implies that the Radon–Nikodym derivative is well-defined in our case. Accordingly, for $s' \in \mathcal{S}^{(t)}$, the Radon–Nikodym derivative is

$$\frac{d\hat{\theta}^{(t)}_{s,a}}{d\hat{\theta}^{(t+1)}_{s,a\to s'}}(s'') = \begin{cases} \frac{\epsilon_{\text{new}} + \epsilon_{\text{known}} |\mathcal{S}^{(t)}| + C^{(t)}_{s,a} + 1}{\epsilon_{\text{new}} + \epsilon_{\text{known}} |\mathcal{S}^{(t)}| + C^{(t)}_{s,a}} & \text{if} \quad s'' \neq s' \,, \\ \frac{\epsilon_{\text{new}} + \epsilon_{\text{known}} |\mathcal{S}^{(t)}| + C^{(t)}_{s,a} + 1}{\epsilon_{\text{new}} + \epsilon_{\text{known}} |\mathcal{S}^{(t)}| + C^{(t)}_{s,a}} \frac{\epsilon_{\text{known}} + C^{(t)}_{s,a,s'}}{\epsilon_{\text{known}} + C^{(t)}_{s,a,s'} + 1} & \text{if} \quad s'' = s' \,. \end{cases} \tag{S41}$$

and for $s' \notin \mathcal{S}^{(t)}$, the Radon–Nikodym derivative is

$$\frac{d\hat{\theta}^{(t)}_{s,a}}{d\hat{\theta}^{(t+1)}_{s,a\to s'}}(s'') = \begin{cases} \frac{\epsilon_{\text{new}} + \epsilon_{\text{known}} |\mathcal{S}^{(t)}| + \epsilon_{\text{known}} + C^{(t)}_{s,a} + 1}{\epsilon_{\text{new}} + \epsilon_{\text{known}} |\mathcal{S}^{(t)}| + C^{(t)}_{s,a}} & \text{if} \quad s'' \neq s' \,, \\ 0 & \text{if} \quad s'' = s' \,. \end{cases} \tag{S42}$$

As a result, the information gain in Equation S39 can be calculated as

$$R_{\text{int},t}(s,a\to s') = \begin{cases} \log\frac{\epsilon_{\text{new}}+\epsilon_{\text{known}}|\mathcal{S}^{(t)}|+C^{(t)}_{s,a}+1}{\epsilon_{\text{new}}+\epsilon_{\text{known}}|\mathcal{S}^{(t)}|+C^{(t)}_{s,a}} + \hat{\theta}^{(t)}_{s,a}(s')\log\frac{\epsilon_{\text{known}}+C^{(t)}_{s,a,s'}}{\epsilon_{\text{known}}+C^{(t)}_{s,a,s'}+1} & \text{if} \quad s'\in\mathcal{S}^{(t)}\,, \\ \log\frac{\epsilon_{\text{new}}+\epsilon_{\text{known}}|\mathcal{S}^{(t)}|+\epsilon_{\text{known}}+C^{(t)}_{s,a}+1}{\epsilon_{\text{new}}+\epsilon_{\text{known}}|\mathcal{S}^{(t)}|+C^{(t)}_{s,a}} & \text{if} \quad s'\notin\mathcal{S}^{(t)}\,, \end{cases} \tag{S43}$$

where we used the fact that $\hat{\theta}^{(t)}_{s,a}(s') = 0$ for a given and fixed $s' \notin \mathcal{S}^{(t)}$.

If $\epsilon_{\text{new}} \to 0$, then the momentary average gain in information after taking action a in state s can be written as

$$\begin{aligned} \bar{IG}^{(t)}(s,a) &:= \mathbb{E}_{S'\sim\hat{\theta}^{(t)}_{s,a}}\Big[IG^{(t)}(s,a\to S')\Big] \\ &= \log\Big[1+\frac{1}{B^{(t)}_{s,a}}\Big] - \sum_{s'\in\mathcal{S}^{(t)}}\Big(\hat{\theta}^{(t)}_{s,a}(s')\Big)^2\log\Big[1+\frac{1}{B^{(t)}_{s,a}\hat{\theta}^{(t)}_{s,a}(s')}\Big], \end{aligned} \tag{S44}$$

where we defined $B^{(t)}_{s,a} := \epsilon_{\text{known}}|\mathcal{S}^{(t)}| + C^{(t)}_{s,a}$. With a few lines of algebra, we can show

$$\frac{\partial \bar{IG}^{(t)}(s,a)}{\partial C^{(t)}_{s,a}} = -\frac{1}{B^{(t)}_{s,a}(1+B^{(t)}_{s,a})}\Big[1 - \sum_{s'\in\mathcal{S}^{(t)}}\Big(\hat{\theta}^{(t)}_{s,a}(s')\Big)^2\frac{1+B^{(t)}_{s,a}}{1+\hat{\theta}^{(t)}_{s,a}(s')B^{(t)}_{s,a}}\Big] \leq 0, \tag{S45}$$

where the equality holds if an only if $\hat{\theta}^{(t)}_{s,a}(s') = 1$ for some s'. Hence, $\bar{IG}^{(t)}(s,a)$ is a decreasing function of the count $C^{(t)}_{s,a}$ of the state-action pair (s,a).

4.4 Seeking surprise or information gain in deterministic environments

Suppose action a in state s is deterministic, meaning that taking a in s will always lead the agent to a specific state s^* with no randomness. If the agent is aware of the deterministic nature of the environment, then taking this action *once* is sufficient to obtain an exact estimate of $p^{(t)}(.|s,a)$. As a result, after trying a deterministic action once, there should intuitively be no remaining surprise or information gain associated with that action. In this section, we show that this intuition is consistent with our formalization of surprise and information gain: The values of surprise and information gain associated with a second try of a deterministic action decrease as a function of the agent's belief in the environment's determinism.

To simplify notation and derivation, we assume $\epsilon_{\text{new}} = 0$ and $\epsilon_{\text{known}} = \epsilon$, though this assumption is not required for the analysis. Combined with the determinism of action a, this allows us to simplify the world model in Equation S7 as

$$p^{(t)}(s'|s,a) = \begin{cases} \frac{\epsilon+\tilde{C}^{(t)}_{s,a}}{N_s\epsilon+\tilde{C}^{(t)}_{s,a}} & \text{if} \quad s'=s^*\,, \\ \frac{\epsilon}{N_s\epsilon+\tilde{C}^{(t)}_{s,a}} & \text{if} \quad s'\neq s^*\,, \end{cases} \tag{S46}$$

where s^* is the next state reached when action a is taken in state s, and we defined $N_s := |\mathcal{S}^{(t)}|$. Following the Bayesian reasoning in Section 4.1, the simplified world model in Equation S46 shows that the agent's belief in the environment's determinism is captured by the magnitude of ϵ. In the limit as $\epsilon \to 0$, a single observation of (s,a) (i.e., $\tilde{C}^{(t)}_{s,a} = 1$) is sufficient for the agent to assign probability 1 to the transition $(s,a) \to s^*$. Furthermore, in this limit, subsequent observations do not lead to an update of the estimated transition probability.

Accordingly, our goal in this section is to examine the theoretical behavior of surprise- and information-gain-seeking strategies in the limit of $\epsilon \to 0$.

Surprise-seeking. For a surprise-seeking agent, the expected reward associated with taking action a in state s is given by

$$\bar{R}_{\text{int},t}(s,a) := \mathbb{E}_{S'\sim\hat{\theta}^{(t)}_{s,a}}\left[R_{\text{int},t}(s,a\to S')\right] = \mathbb{E}_{S'\sim\hat{\theta}^{(t)}_{s,a}}\left[-\log p^{(t)}(S'|s,a)\right]. \tag{S47}$$

Using Equation S46, it is straightforward to see that, before trying action a, the average reward associated with a is independent of the agent's belief about the environment's determinism (i.e., ϵ):

$$\tilde{C}^{(t)}_{s,a} = 0 \quad \Rightarrow \quad \bar{R}_{\text{int},t}(s,a) = \log N_s. \tag{S48}$$

However, as soon as $\tilde{C}^{(t)}_{s,a} > 0$, the average reward drastically decreases, i.e.,

$$\begin{aligned}&\tilde{C}^{(t)}_{s,a} \geq 1 \gg \epsilon \quad \Rightarrow \\ &\bar{R}_{\text{int},t}(s,a) = -(1-\tilde{\epsilon})\log(1-\tilde{\epsilon}) - \tilde{\epsilon}\log\frac{\tilde{\epsilon}}{N_s-1} = \mathcal{O}\big(\epsilon\log\epsilon\big)\end{aligned} \tag{S49}$$

where we defined

$$\tilde{\epsilon} := \frac{(N_s-1)\epsilon}{N_s\epsilon + \tilde{C}^{(t)}_{s,a}}. \tag{S50}$$

Accordingly, a surprise-seeking agent that believes the environment is deterministic will experience a sharp decrease in the intrinsic reward of a deterministic action after trying it once. Intuitively, this means that such an agent will aim to try as many actions as possible in a deterministic environment.

Information-gain-seeking. For an information-gain-seeking agent, the expected reward associated with taking action a in state s is given by

$$\bar{R}_{\text{int},t}(s,a) := \mathbb{E}_{S'\sim\hat{\theta}^{(t)}_{s,a}}\left[R_{\text{int},t}(s,a\to S')\right] \tag{S51}$$

with

$$R_{\text{int},t}(s,a\to s') = \begin{cases} \log\frac{\epsilon N_s+\tilde{C}^{(t)}_{s,a}+1}{\epsilon N_s+\tilde{C}^{(t)}_{s,a}} + \frac{\epsilon+\tilde{C}^{(t)}_{s,a}}{N_s\epsilon+\tilde{C}^{(t)}_{s,a}}\log\frac{\epsilon+\tilde{C}^{(t)}_{s,a}}{\epsilon+\tilde{C}^{(t)}_{s,a}+1} & \text{if} \quad s'=s^*, \\ \log\frac{\epsilon N_s+\tilde{C}^{(t)}_{s,a}+1}{\epsilon N_s+\tilde{C}^{(t)}_{s,a}} + \frac{\epsilon}{N_s\epsilon+\tilde{C}^{(t)}_{s,a}}\log\frac{\epsilon}{\epsilon+1} & \text{if} \quad s'\neq s^*, \end{cases} \tag{S52}$$

where we used Equation S43 and Equation S46 to evaluate $R_{\text{int},t}(s,a\to s')$.

Intriguingly, before trying action a, the average reward associated with a increases as the agent's belief about the environment's determinism increases (as $\epsilon\to 0$), i.e.,

$$\tilde{C}^{(t)}_{s,a} = 0 \quad \Rightarrow \quad \bar{R}_{\text{int},t}(s,a) = \log\left(1+\frac{1}{N_s\epsilon}\right) - \frac{1}{N_s}\log\left(1+\frac{1}{\epsilon}\right) = \mathcal{O}\big(-\log\epsilon\big). \tag{S53}$$

Intuitively, this implies that, as the agent's belief in the environment's determinism increases (as $\epsilon\to 0$), the agent becomes increasingly confident that taking a single action will resolve the whole uncertainty about the transition probabilities, resulting in a substantial gain of information. Asymptotically, if $\tilde{C}^{(t)}_{s,a} = 0$, then we have $\lim_{\epsilon\to 0}\bar{R}_{\text{int},t}(s,a) = \infty$.

However, similarly to the case of surprise, only taking action a once drastically decreases its associated

intrinsic rewards, i.e.,

$$\tilde{C}^{(t)}_{s,a} \geq 1 \gg \epsilon \Rightarrow$$

$$\bar{R}_{\text{int},t}(s,a) = \log \frac{\epsilon N_s + \tilde{C}^{(t)}_{s,a} + 1}{\epsilon N_s + \tilde{C}^{(t)}_{s,a}} + \left(\frac{\epsilon + \tilde{C}^{(t)}_{s,a}}{N_s\epsilon + \tilde{C}^{(t)}_{s,a}}\right)^2 \log \frac{\epsilon + \tilde{C}^{(t)}_{s,a}}{\epsilon + \tilde{C}^{(t)}_{s,a} + 1} + \frac{N_s\epsilon^2}{\left(N_s\epsilon + \tilde{C}^{(t)}_{s,a}\right)^2} \log \frac{\epsilon}{\epsilon + 1} = \mathcal{O}(\epsilon). \tag{S54}$$

Accordingly, an information-gain-seeking agent that believes the environment is deterministic will aim to try as many actions as possible in a deterministic environment. As a result, seeking information-gain and seeking surprise are not behaviorally dissociable in these situations.

4.5 Analysis of the MB optimistic initialization in episode 2

To theoretically analyze the influence of the MB optimistic initialization in episode 2, we make a few simplistic assumptions:

1. ϵ_{new} in Equation S29 is negligible.
2. All transition probabilities except for the ones between the stochastic states and the progressing action in state 6 (because of the only one-time experience) have been learned with certainty during the 1st episode.
3. The counts for the actions in the stochastic part are roughly the same for all states and actions, which we denote by $\bar{C}^{(t)}$, i.e., for any state s_s in the stochastic part, we assume that $C^{(t)}_{s_s,a} = \bar{C}^{(t)}$ for every action a.

Given these assumptions, we have a symmetry between the stochastic states, implying that the Q-values in the stochastic part are the same for all states. Hence, in all the following equations, we use s_s to denote a representative state in the stochastic part, use a_p to refer to the progressing actions, use a_s to refer to the stochastic/self-looping actions, denote state 4 by $s4$, denote state 6 by $s6$, use r_{G^*} to denote the reward value of the already discovered goal, and define

$$\bar{R} := \frac{1}{|\mathcal{S}^{(t)}|}\left(1 + r^*_1 + r^*_2\right) \quad \text{and} \quad \bar{V}^{(t)} := \frac{1}{|\mathcal{S}^{(t)}|}\sum_{s'} V^{(t)}_{\text{MB,ext}}(s'). \tag{S55}$$

Using these notations and assumptions as well as Equation S29 and Equation S32, we have

$$\begin{aligned} Q^{(t)}_{\text{MB,ext}}(s_s,a_p) &= p^{(t)}_s\left(\bar{R} + \gamma_{\text{ext}}\bar{V}^{(t)}\right) + \gamma_{\text{ext}}\left(1 - p^{(t)}_s\right)V^{(t)}_{\text{MB,ext}}(s4) \\ Q^{(t)}_{\text{MB,ext}}(s_s,a_s) &= p^{(t)}_s\left(\bar{R} + \gamma_{\text{ext}}\bar{V}^{(t)}\right) + \gamma_{\text{ext}}\left(1 - p^{(t)}_s\right)V^{(t)}_{\text{MB,ext}}(s_s), \end{aligned} \tag{S56}$$

where

$$p^{(t)}_s = \frac{\epsilon_{\text{known}}|\mathcal{S}^{(t)}|}{\epsilon_{\text{known}}|\mathcal{S}^{(t)}| + \bar{C}^{(t)}}. \tag{S57}$$

Note that $\frac{p^{(t)}_s}{|\mathcal{S}^{(t)}|}$ is equal to the probability of transition to any state s' for which $C_{s_s,a,s'} = 0$ (see Equation S29).

If the optimal policy is to leave the stochastic part and go to the already discovered goal state, then we must have

$$\text{Condition 1: } Q^{(t)}_{\text{MB,ext}}(s_s,a_s) < Q^{(t)}_{\text{MB,ext}}(s_s,a_p) = V^{(t)}_{\text{MB,ext}}(s_s). \tag{S58}$$

According to Equation S56, Condition 1 is equivalent to $Q^{(t)}_{\text{MB,ext}}(s_s, a_p) = V^{(t)}_{\text{MB,ext}}(s_s) \leq V^{(t)}_{\text{MB,ext}}(s4)$, which, by using Equation S56 again and after a few lines of algebra, can be written as

$$\text{Condition 1} \equiv \frac{p_s^{(t)}}{1 - \gamma_{\text{ext}}(1 - p_s^{(t)})} \Big[\bar{R} + \gamma_{\text{ext}} \bar{V}^{(t)}\Big] < V^{(t)}_{\text{MB,ext}}(s4). \tag{S59}$$

Given that the optimal policy under Condition 1 is to leave the stochastic part and go to the already discovered goal state, we can write the value of state 4 as

$$V^{(t)}_{\text{MB,ext}}(s4) = Q^{(t)}_{\text{MB,ext}}(s4, a_p) = \gamma^2_{\text{ext}} Q^{(t)}_{\text{MB,ext}}(s6, a_p) = \gamma^2_{\text{ext}} \Big[\tilde{p}_g^{(t)}\big(\bar{R} + \gamma_{\text{ext}}\bar{V}^{(t)}\big) + \big(1 - p_g^{(t)}\big) r_{G^*}\Big], \tag{S60}$$

where

$$p_g^{(t)} = \frac{\epsilon_{\text{known}} |\mathcal{S}^{(t)}|}{\epsilon_{\text{known}} |\mathcal{S}^{(t)}| + 1} \quad \text{and} \quad \tilde{p}_g^{(t)} = p_g^{(t)} + \gamma_{\text{ext}}(1 - p_g^{(t)}). \tag{S61}$$

Note that $\frac{p_g^{(t)}}{|\mathcal{S}^{(t)}|}$ is equal to the probability of transition to any state s' for which $C_{s6,a_p,s'} = 0$ (see Equation S29). Using Equation S60, we can simplify Equation S59 as

$$\text{Condition 1} \equiv f_{\text{C1}}(r_1^*, r_2^*, \gamma_{\text{ext}}, \epsilon_{\text{known}}, \bar{C}^{(t)}, |\mathcal{S}^{(t)}|) < r_{G^*}, \tag{S62}$$

with

$$f_{\text{C1}}(r_1^*, r_2^*, \gamma_{\text{ext}}, \epsilon_{\text{known}}, \bar{C}^{(t)}, |\mathcal{S}^{(t)}|) := \frac{1}{\gamma^2_{\text{ext}}(1 - p_g^{(t)})} \Big[\frac{p_s^{(t)}}{1 - \gamma_{\text{ext}}(1 - p_s^{(t)})} - \gamma^2_{\text{ext}} \tilde{p}_g^{(t)}\Big] \Big[\bar{R} + \gamma_{\text{ext}} \bar{V}^{(t)}\Big]. \tag{S63}$$

The variable $R^{(t)}_{\text{Stoch.}}$ in Section 2.2 is $\gamma^2_{\text{ext}} f_{\text{C1}}$.

An important observation is that, independently of the parameter values, we have

$$\lim_{\bar{C}^{(t)} \to \infty} f_{\text{C1}}(r_1^*, r_2^*, \gamma_{\text{ext}}, \epsilon_{\text{known}}, \bar{C}^{(t)}, |\mathcal{S}^{(t)}|) < 0.$$

This implies that, for any value of r_2^* and $r_{G^*} > 0$, increasing $\bar{C}^{(t)}$ would eventually result in a preference for leaving the stochastic part and going towards the already discovered goal (Condition 1 is satisfied). In other words, agents will eventually give up exploration after a sufficiently long and unsuccessful exploration phase. This is why the MB optimistic initialization is similar to exploration driven by information gain.

Moreover, by analyzing f_{C1}, we can gain further insights about how the model parameters influence exploration based on the MB optimistic initialization:

1. For any value of r_2^* and r_{G^*}, we have

   $$\lim_{\gamma_{\text{ext}} \to 0} f_{\text{C1}}(r_1^*, r_2^*, \gamma_{\text{ext}}, \epsilon_{\text{known}}, \bar{C}^{(t)}, |\mathcal{S}^{(t)}|) = \infty.$$

   This implies that decreasing the discount factor to put a small weight on the future rewards would make the agent stay in the stochastic part (Condition 1 is violated).

2. If $r_{G^*} < r_2^*$ (i.e., the agent knows that there exists a goal state with a reward higher than the one already discovered) and $\gamma^2_{\text{ext}} \tilde{p}_g^{(t)} < \frac{p_s^{(t)}}{1 - \gamma_{\text{ext}}(1 - p_s^{(t)})}$ (i.e., the discount factor is small enough; see point 1), then we have

   $$\lim_{r_2^* \to \infty} f_{\text{C1}}(r_1^*, r_2^*, \gamma_{\text{ext}}, \epsilon_{\text{known}}, \bar{C}^{(t)}, |\mathcal{S}^{(t)}|) > r_{G^*}.$$

This implies that, if $r_{G^*} < r_2^*$, then increasing r_2^* would eventually result in a preference for staying in the stochastic part (Condition 1 is violated). In other words, if the reward value of one of the three goal states is much greater than the discovered goal state, then the agent prefers to keep exploring the stochastic part.

3. For any value of r_2^* and r_{G^*}, we have

$$\lim_{\epsilon_{\text{known}} \to 0} f_{\text{C1}}(r_1^*, r_2^*, \gamma_{\text{ext}}, \epsilon_{\text{known}}, \bar{C}^{(t)}, |\mathcal{S}^{(t)}|) < 0.$$

This implies that, independently of the reward value of the discovered goal state, if the agent assigns a very small prior probability to the unseen transitions, then the agent always prefers to leave the stochastic part and go to the already discovered goal state (i.e., Condition 1 is satisfied).

4.6 Optimistic initialization in tandem with intrinsic rewards

When presenting different exploration strategies in Section 2.3, we mentioned that we set $\beta^{(1)}_{\text{MF,ext}} = \beta^{(1)}_{\text{MB,ext}} = 0$ and $Q^{(0)}_{\text{MF,ext}} = 0$ to dissociate the effect of exploration driven by intrinsic rewards from that driven by optimistic initialization in *episode 1*. In this section, we further elaborate on this choice and discuss the interplay between intrinsic rewards and optimistic initialization in our models.

Specifically, we emphasize that this parameter setting (i.e., $\beta^{(1)}_{\text{MF,ext}} = \beta^{(1)}_{\text{MB,ext}} = 0$ and $Q^{(0)}_{\text{MF,ext}} = 0$) removes the effect of optimistic initialization *only* from the extrinsic-reward-seeking component and *only* during episode 1. This has two important implications. First, the mechanism for MB optimistic initialization described in Section 2.2 can still contribute to directed exploration *during episodes 2–5*. In this sense, models with intrinsic rewards include two parallel mechanisms for balancing exploration and exploitation, both controlled by the extent of the 'reward optimism.' Second, even during episode 1, the choices of $Q^{(0)}_{\text{MF,int}}$, ϵ_{known}, and ϵ_{new} result in an optimistic initialization of the *intrinsic value* for unobserved states and actions.

In other words, optimistic initialization and intrinsic-reward-seeking operate in tandem in our intrinsically motivated RL algorithms. From this perspective, the poor performance of nRI in our work (Figure 4) should not be taken as evidence that humans do not employ optimistic initialization to explore their environment. Rather, our results indicate that a form of optimistic initialization limited to extrinsic rewards alone is insufficient to explain participants' behavior.

5 Supplementary Methods: Algorithmic implementation

5.1 Initialization

For Epi > 1, $\mathcal{S}^{(0)}$, $\tilde{C}^{(0)}$, $U^{(0)}_{\text{ext}}$, $U^{(0)}_{\text{int}}$, $Q^{(0)}_{\text{MB,ext}}$, $Q^{(0)}_{\text{MB,int}}$, $Q^{(0)}_{\text{MF,ext}}$, and $Q^{(0)}_{\text{MF,int}}$ are initialized by their latest value in the previous episode.

For Epi $= 1$, the initial values are as follows:

$$\begin{aligned} \mathcal{S}^{(0)} &= \{G_0, G_1, G_2\}, \\ \tilde{C}^{(0)} &= 0, \\ Q^{(0)}_{\text{MF,ext}}(s,a) &= Q^{(0)}_{\text{MF,ext}}, \\ Q^{(0)}_{\text{MF,int}}(s,a) &= Q^{(0)}_{\text{MF,int}}. \end{aligned} \tag{S64}$$

For the model-based Q-values, we can analytically solve the Bellman equations at time $t = 0$, resulting in

$$\begin{aligned} &U^{(0)}_{\text{ext}}(s) = Q^{(0)}_{\text{MB,ext}}(s,a) = \frac{\hat{\theta}_{\text{known}} + \gamma_{\text{ext}}\hat{\theta}_{\text{new}}W_{\text{known}}}{1 - \gamma_{\text{ext}}|\mathcal{S}^{(0)}|\Big(\hat{\theta}_{\text{known}} + \gamma_{\text{ext}}\hat{\theta}_{\text{new}}W_{\text{known}}\Big)}(1 + r_1 + r_2), \\ &U^{(0)}_{\text{int}}(s) = Q^{(0)}_{\text{MB,int}}(s,a) \\ &= \frac{\hat{\theta}_{\text{known}}|\mathcal{S}^{(0)}|R^{(\text{int})}_{\text{known}}(s_{\text{known}}) + \hat{\theta}_{\text{new}}\Big(R^{(\text{int})}_{\text{known}}(s_{\text{new}}) + \gamma_{\text{int}}W_{\text{new}}R^{(\text{int})}_{\text{new}}(s_{\text{new}}) + |\mathcal{S}^{(0)}|\gamma_{\text{int}}W_{\text{known}}R^{(\text{int})}_{\text{new}}(s_{\text{known}})\Big)}{1 - \gamma_{\text{int}}|\mathcal{S}^{(0)}|\Big(\hat{\theta}_{\text{known}} + \gamma_{\text{int}}\hat{\theta}_{\text{new}}W_{\text{known}}\Big)}, \end{aligned} \tag{S65}$$

with

$$\begin{aligned} W_{\text{known}} &= \frac{\epsilon_{\text{known}}}{(1-\gamma)\epsilon_{\text{new}} + \epsilon_{\text{known}}|\mathcal{S}^{(0)}|} \quad , \quad W_{\text{new}} = \frac{\epsilon_{\text{new}}}{(1-\gamma)\epsilon_{\text{new}} + \epsilon_{\text{known}}|\mathcal{S}^{(0)}|}, \\ \hat{\theta}_{\text{known}} &= \frac{\epsilon_{\text{known}}}{\epsilon_{\text{new}} + \epsilon_{\text{known}}|\mathcal{S}^{(0)}|} \quad , \quad \hat{\theta}_{\text{new}} = \frac{\epsilon_{\text{new}}}{\epsilon_{\text{new}} + \epsilon_{\text{known}}|\mathcal{S}^{(0)}|} \end{aligned} \tag{S66}$$

and

$$\begin{aligned} R^{(\text{int})}_{\text{new}}(s_{\text{known}}) &= R_{\text{int},0}(s_{\text{new}}, a \to s) \quad , \quad R^{(\text{int})}_{\text{new}}(s_{\text{new}}) = R_{\text{int},0}(s_{\text{new}}, a \to s_{\text{new}}) \\ R^{(\text{int})}_{\text{known}}(s_{\text{known}}) &= R_{\text{int},0}(s, a \to s) \quad , \quad R^{(\text{int})}_{\text{known}}(s_{\text{new}}) = R_{\text{int},0}(s, a \to s_{\text{new}}) \end{aligned} \tag{S67}$$

for any $a \in \mathcal{A}$ and $s \in \mathcal{S}^{(0)}$.

However, the final (after learning the transition probabilities) values for $Q_{\text{MB,ext}}(s,a)$ are much smaller than the analytic solution to the Bellman equation at $t = 0$—due to the sparse connections and a single path to one goal state. We, therefore, use a heuristic and put $U^{(0)}_{\text{ext}}(s) = Q^{(0)}_{\text{MB,ext}}(s,a) = 0$.

5.2 Pseudocode

See Algorithms 1, 2, 3, and 4 for pseudocode. Note that, in all pseudocode, we use an alternative shorter notation by defining $R^{(\text{int},t)}_{s,a}(s') := R_{\text{int},t}(s, a \to s')$ and $R^{(\text{ext})}_{s,a}(s') := R_{\text{ext}}(s, a \to s')$.

Algorithm 1 General pseudocode for algorithm

\# Setting specification

Specify
$$\Phi = \{\Phi^{(\text{main})}, \Phi^{(\beta)}, \Phi^{(b)}\}$$

Specify the intrinsic reward function $R_{s,a}^{(\text{int},t)}(s')$.

Specify Episode (Epi) and the set of possible actions $\mathcal{A}$.

if Epi $= 1$ **then**

  $\beta_{\text{MB,ext}} \leftarrow \beta^{(1)}_{\text{MB,ext}}$, $\beta_{\text{MF,ext}} \leftarrow \beta^{(1)}_{\text{MF,ext}}$, $\beta_{\text{MB,int}} \leftarrow \beta^{(1)}_{\text{MB,int}}$, and $\beta_{\text{MF,int}} \leftarrow \beta^{(1)}_{\text{MF,int}}$.

else

  $\beta_{\text{MB,ext}} \leftarrow \beta^{(2,r_{G^*})}_{\text{MB,ext}}$, $\beta_{\text{MF,ext}} \leftarrow \beta^{(2,r_{G^*})}_{\text{MF,ext}}$, $\beta_{\text{MB,int}} \leftarrow \beta^{(2,r_{G^*})}_{\text{MB,int}}$, and $\beta_{\text{MF,int}} \leftarrow \beta^{(2,r_{G^*})}_{\text{MF,int}}$.

end if

\# Initialization (all variables are defined only for $s \in \mathcal{S}^{(0)}$)

Initialize $\mathcal{S}^{(0)}$, $\tilde{C}^{(0)}$, $U^{(0)}_{\text{ext}}$, $U^{(0)}_{\text{int}}$, $Q^{(0)}_{\text{MB,ext}}$, $Q^{(0)}_{\text{MB,int}}$, $Q^{(0)}_{\text{MF,ext}}$, and $Q^{(0)}_{\text{MF,int}}$ (cf. Section 5.1).

\# 1st observation

$t \leftarrow 0$

Initialize state s_1 and update $\tilde{C}^{(1)}_s \leftarrow \kappa \tilde{C}^{(0)}_s + \delta_{s,s_1}$ and $\mathcal{S}^{(1)} \leftarrow \mathcal{S}^{(0)} \cup \{s_1\}$.

$\tilde{C}^{(1)}_{s,a,s'} \leftarrow \tilde{C}^{(0)}_{s,a,s'}$ for all s and $s' \in \mathcal{S}^{(0)}$.

$Q^{(1)}_{\text{MF,ext}}(s,a) \leftarrow Q^{(0)}_{\text{MF,ext}}(s,a)$ and $Q^{(1)}_{\text{MF,int}}(s,a) \leftarrow Q^{(0)}_{\text{MF,int}}(s,a)$ for all $s \in \mathcal{S}^{(0)}$.

$e^{(1)}_{\text{ext}} \leftarrow 0$ and $e^{(1)}_{\text{int}} \leftarrow 0$.

\# Extensions of variables for $s_1 \notin \mathcal{S}^{(0)}$

$\tilde{C}^{(1)}_{s,a,s'} \leftarrow 0$ if $s = s_1$ or $s' = s_1$ and $s_1 \notin \mathcal{S}^{(0)}$.

$Q^{(1)}_{\text{MF,ext}}(s_1,a) \leftarrow Q^{(0)}_{\text{MF,ext}}$ and $Q^{(1)}_{\text{MF,int}}(s_1,a) \leftarrow Q^{(0)}_{\text{MF,int}}$ if $s_1 \notin \mathcal{S}^{(0)}$.

Update $U^{(1)}_{\text{ext}}$, $U^{(1)}_{\text{int}}$, $Q^{(1)}_{\text{MB,ext}}$, and $Q^{(1)}_{\text{MB,int}}$ using the model-based branch in Alg. 2.

\# Going through the task

$t \leftarrow 1$.

while $s_t \neq G_i$ for $i \in \{0,1,2\}$ **do**

\# Making action

  Compute $Q^{(t)}_{\text{MF}}(s,a) \leftarrow \beta_{\text{MF,ext}} Q^{(t)}_{\text{MF,ext}}(s,a) + \beta_{\text{MF,int}} Q^{(t)}_{\text{MF,int}}(s,a)$.

  Compute $Q^{(t)}_{\text{MB}}(s,a) \leftarrow \beta_{\text{MB,ext}} Q^{(t)}_{\text{MB,ext}}(s,a) + \beta_{\text{MB,int}} Q^{(t)}_{\text{MB,int}}(s,a)$.

  Sample a_t with probability $\pi(a_t|s_t) \propto \exp\left\{Q^{(t)}_{\text{MF}}(s_t,a_t) + Q^{(t)}_{\text{MB}}(s_t,a_t) + b(a_t)\right\}$.

  Observe s_{t+1}.

\# Updating internal variables

  $\mathcal{S}^{(t+1)} \leftarrow \mathcal{S}^{(t)} \cup \{s_{t+1}\}$.

  Update counts $\tilde{C}^{(t+1)}_s \leftarrow \kappa \tilde{C}^{(t)}_s + \delta_{s,s_{t+1}}$ and $\tilde{C}^{(t+1)}_{s_t,a_t,s'} \leftarrow \kappa \tilde{C}^{(t)}_{s_t,a_t,s'} + \delta_{s',s_{t+1}}$.

  $\tilde{C}^{(t+1)}_{s,a,s'} \leftarrow \tilde{C}^{(t)}_{s,a,s'}$ if $s \neq s_t$ or $a \neq a_t$.

  Update $U^{(t+1)}_{\text{ext}}$, $U^{(t+1)}_{\text{int}}$, $Q^{(t+1)}_{\text{MB,ext}}$, and $Q^{(t+1)}_{\text{MB,int}}$ using the model-based branch in Alg. 2.

  Update $e^{(t+1)}_{\text{ext}}$, $e^{(t+1)}_{\text{int}}$, $Q^{(t+1)}_{\text{MF,ext}}$, and $Q^{(t+1)}_{\text{MF,int}}$ using the model-free branch in Alg. 3.

\# Going to the next step

  $t \leftarrow t + 1$.

end while

Algorithm 2 Pseudocode for the model-based branch

$\tilde{C}_{s,a}^{(t+1)} \leftarrow \sum_{s'} \tilde{C}_{s,a,s'}^{(t+1)}$.

Updating the world model

$\hat{\theta}_{s,a}^{(t+1)}(s') \leftarrow \big(\epsilon_{\text{known}} + \tilde{C}_{s,a,s'}^{(t+1)}\big)/\big(\epsilon_{\text{new}} + \epsilon_{\text{known}}|\mathcal{S}^{(t+1)}| + \tilde{C}_{s,a}^{(t+1)}\big)$ for $s' \in \mathcal{S}^{(t+1)}$.

$\hat{\theta}_{s,a}^{(t+1)}(s_{\text{new}}) \leftarrow \big(\epsilon_{\text{new}}\big)/\big(\epsilon_{\text{new}} + \epsilon_{\text{known}}|\mathcal{S}^{(t+1)}| + \tilde{C}_{s,a}^{(t+1)}\big)$.

Updating the values

Update $Q_{\text{MB,int}}^{(t+1)}$ and $U_{\text{int}}^{(t+1)}$ using Alg. 4 and $R^{(\text{int},t+1)}$ as rewards.

Update $Q_{\text{MB,ext}}^{(t+1)}$ and $U_{\text{ext}}^{(t+1)}$ using Alg. 4 and $R^{(\text{ext})}$ as rewards.

Algorithm 3 Pseudocode for the model-free branch

Prediction errors

$RPE_{\text{ext},t+1} \leftarrow R_{s_t,a_t}^{(\text{ext})}(s_{t+1}) + \gamma_{\text{ext}} \max_{a' \in \mathcal{A}} Q_{\text{MF,ext}}^{(t)}(s_{t+1}, a') - Q_{\text{MF,ext}}^{(t)}(s_t, a_t)$.

$RPE_{\text{int},t+1} \leftarrow R_{s_t,a_t}^{(\text{int},t)}(s_{t+1}) + \gamma_{\text{int}} \max_{a' \in \mathcal{A}} Q_{\text{MF,int}}^{(t)}(s_{t+1}, a') - Q_{\text{MF,int}}^{(t)}(s_t, a_t)$.

Update of the eligibility traces

$e_{\text{ext}}^{(t+1)}(s_t, a_t) \leftarrow 1$, and $e_{\text{ext}}^{(t+1)}(s, a) \leftarrow \gamma_{\text{ext}}\lambda_{\text{ext}} e_{\text{ext}}^{(t)}(s, a)$, for all $s \neq s_t$ and $a \neq a_t$.

$e_{\text{int}}^{(t+1)}(s_t, a_t) \leftarrow 1$, and $e_{\text{int}}^{(t+1)}(s, a) \leftarrow \gamma_{\text{int}}\lambda_{\text{int}} e_{\text{int}}^{(t)}(s, a)$, for all $s \neq s_t$ and $a \neq a_t$.

TD-learners

$Q_{\text{MF,ext}}^{(t+1)}(s, a) \leftarrow Q_{\text{MF,ext}}^{(t)}(s, a) + \rho e_{\text{ext}}^{(t+1)}(s, a) RPE_{\text{ext},t+1}$, $\forall s \in \mathcal{S}$ and $a \in \mathcal{A}$.

$Q_{\text{MF,int}}^{(t+1)}(s, a) \leftarrow Q_{\text{MF,int}}^{(t)}(s, a) + \rho e_{\text{int}}^{(t+1)}(s, a) RPE_{\text{int},t+1}$, $\forall s \in \mathcal{S}$ and $a \in \mathcal{A}$.

Algorithm 4 Pseudocode for the modified Prioritized Sweeping Algorithm at time $t+1$

\# Specifying whether the update is for the intrinsic or the extrinsic reward

$\gamma \leftarrow \gamma_{\text{ext}}$ for extrinsic and $\gamma \leftarrow \gamma_{\text{int}}$ for intrinsic reward.

$Q^{(t)} \leftarrow Q^{(t)}_{\text{MB,ext}}$, $U^{(t)} \leftarrow U^{(t)}_{\text{ext}}$, and $R \leftarrow R^{(\text{ext})}$ for extrinsic, and $Q^{(t)} \leftarrow Q^{(t)}_{\text{MB,int}}$, $U^{(t)} \leftarrow U^{(t)}_{\text{int}}$, and $R \leftarrow R^{(\text{int},t+1)}$ for intrinsic reward.

\# Extending U-values

$W_{\text{known}} \leftarrow \epsilon_{\text{known}} / \big((1-\gamma)\epsilon_{\text{new}} + \epsilon_{\text{known}}|\mathcal{S}^{(t+1)}|\big)$ and $W_{\text{new}} \leftarrow \epsilon_{\text{new}} / \big((1-\gamma)\epsilon_{\text{new}} + \epsilon_{\text{known}}|\mathcal{S}^{(t+1)}|\big)$

if $s_{t+1} \notin \mathcal{S}^{(t)}$ **then**

  $U^{(t)}(s_{t+1}) \leftarrow W_{\text{known}} \sum_{s' \in \mathcal{S}^{(t)}} \Big(R_{s_{\text{new}}}(s') + \gamma U^{(t)}(s')\Big) + W_{\text{new}} R_{s_{\text{new}}}(s_{\text{new}})$.

end if

\# Applying the effect of the latest observation on Q-values using previous U-values

for $(s,a) \in \mathcal{S}^{(t+1)} \times \mathcal{A}$ **do**

  $Q^{(t+1)}(s,a) \leftarrow Q\text{Update}(s,a;\gamma,\hat{\theta}^{(t+1)},U^{(t)},R,W_{\text{known}},W_{\text{new}},\mathcal{S}^{(t+1)})$ defined in Eq. S38.

end for

\# Making the priority queue

for $s \in \mathcal{S}^{(t+1)}$ **do**

  $U^{(t+1)}(s) \leftarrow U^{(t)}(s)$

  $\text{PriorityQueue}(s) \leftarrow |U^{(t+1)}(s) - \max_{a\in\mathcal{A}} Q^{(t+1)}(s,a)|$

end for

\# Updating U-values for T_{PS} steps

for T_{PS} iterations **do**

  $s' \leftarrow \arg\max_{s\in\mathcal{S}^{(t+1)}} \text{PriorityQueue}(s)$

  $\Delta V \leftarrow \max_{a\in\mathcal{A}} Q^{(t+1)}(s',a) - U^{(t+1)}(s')$

  $U^{(t+1)}(s') \leftarrow \max_{a\in\mathcal{A}} Q^{(t+1)}(s',a)$

  \# Applying the effect of the update of U-values on Q-values

  for $(s,a) \in \mathcal{S}^{(t+1)} \times \mathcal{A}$ **do**

    $Q^{(t+1)}(s,a) \leftarrow Q^{(t+1)}(s,a) + \gamma\Big(\hat{\theta}^{(t+1)}_{s,a}(s') + \gamma\hat{\theta}^{(t+1)}_{s,a}(s_{\text{new}})W_{\text{known}}\Big)\Delta V$

  end for

  \# Updating the priority queue

  for $s \in \mathcal{S}^{(t+1)}$ **do**

    $\text{PriorityQueue}(s) \leftarrow |U^{(t+1)}(s) - \max_{a\in\mathcal{A}} Q^{(t+1)}(s,a)|$

  end for

end for

References

[1] Ghahramani, Z. Bayesian non-parametrics and the probabilistic approach to modelling. *Philosophical Transactions of the Royal Society A: Mathematical, Physical and Engineering Sciences* **371**, 20110553 (2013).

[2] Xu, H. A., Modirshanechi, A., Lehmann, M. P., Gerstner, W. & Herzog, M. H. Novelty is not surprise: Human exploratory and adaptive behavior in sequential decision-making. *PLoS Computational Biology* **17** (2021).

[3] Liakoni, V. *et al.* Brain signals of a surprise-actor-critic model: Evidence for multiple learning modules in human decision making. *NeuroImage* **246**, 118780 (2022).

[4] Gläscher, J., Daw, N., Dayan, P. & O'Doherty, J. P. States versus rewards: Dissociable neural prediction error signals underlying model-based and model-free reinforcement learning. *Neuron* **66**, 585–595 (2010).

[5] Daw, N., Gershman, S., Seymour, B., Dayan, P. & Dolan, R. Model-based influences on humans' choices and striatal prediction errors. *Neuron* **69**, 1204–1215 (2011).

[6] Sutton, R. S. & Barto, A. G. *Reinforcement learning: An introduction* (MIT press, 2018).

[7] Lehmann, M. P. *et al.* One-shot learning and behavioral eligibility traces in sequential decision making. *eLife* **8**, e47463 (2019).

[8] Yu, A. J. & Cohen, J. D. Sequential effects: Superstition or rational behavior? In Koller, D., Schuurmans, D., Bengio, Y. & Bottou, L. (eds.) *Advances in Neural Information Processing Systems*, vol. 21 (Curran Associates, Inc., 2009).

[9] Liakoni, V., Modirshanechi, A., Gerstner, W. & Brea, J. Learning in volatile environments with the Bayes factor surprise. *Neural Computation* **33**, 1–72 (2021).

[10] Van Seijen, H. & Sutton, R. Planning by prioritized sweeping with small backups. In Dasgupta, S. & McAllester, D. (eds.) *Proceedings of the 30th International Conference on Machine Learning*, vol. 28 of *Proceedings of Machine Learning Research*, 361–369 (PMLR, Atlanta, Georgia, USA, 2013).

[11] Huys, Q. J. *et al.* Interplay of approximate planning strategies. *Proceedings of the National Academy of Sciences* **112**, 3098–3103 (2015).

[12] Piray, P. & Daw, N. D. Linear reinforcement learning in planning, grid fields, and cognitive control. *Nature Communications* **12**, 4942 (2021).

[13] Kolter, J. Z. & Ng, A. Y. Near-Bayesian exploration in polynomial time. In *Proceedings of the 26th Annual International Conference on Machine Learning*, ICML '09, 513–520 (Association for Computing Machinery, New York, NY, USA, 2009).

[14] Aubret, A., Matignon, L. & Hassas, S. An information-theoretic perspective on intrinsic motivation in reinforcement learning: A survey. *Entropy* **25** (2023).

[15] Ladosz, P., Weng, L., Kim, M. & Oh, H. Exploration in deep reinforcement learning: A survey. *Information Fusion* **85**, 1–22 (2022).

[16] Modirshanechi, A., Kondrakiewicz, K., Gerstner, W. & Haesler, S. Curiosity-driven exploration: foundations in neuroscience and computational modeling. *Trends in Neurosciences* **46**, 1054–1066 (2023).

[17] Bellemare, M. *et al.* Unifying count-based exploration and intrinsic motivation. In Lee, D., Sugiyama,

M., Luxburg, U., Guyon, I. & Garnett, R. (eds.) *Advances in Neural Information Processing Systems*, vol. 29 (Curran Associates, Inc., 2016).

[18] Ostrovski, G., Bellemare, M. G., van den Oord, A. & Munos, R. Count-based exploration with neural density models. In *Proceedings of the 34th International Conference on Machine Learning - Volume 70*, ICML'17, 2721–2730 (JMLR.org, 2017).

[19] Cover, T. M. *Elements of information theory* (John Wiley & Sons, 1999).

[20] Mobin, S. A., Arnemann, J. A. & Sommer, F. Information-based learning by agents in unbounded state spaces. In Ghahramani, Z., Welling, M., Cortes, C., Lawrence, N. & Weinberger, K. Q. (eds.) *Advances in Neural Information Processing Systems*, vol. 27 (Curran Associates, Inc., 2014).

[21] Kolossa, A., Kopp, B. & Fingscheidt, T. A computational analysis of the neural bases of Bayesian inference. *NeuroImage* **106**, 222–237 (2015).

[22] Modirshanechi, A., Brea, J. & Gerstner, W. A taxonomy of surprise definitions. *Journal of Mathematical Psychology* **110**, 102712 (2022).

[23] Pathak, D., Gandhi, D. & Gupta, A. Self-supervised exploration via disagreement. In Chaudhuri, K. & Salakhutdinov, R. (eds.) *Proceedings of the 36th International Conference on Machine Learning*, vol. 97 of *Proceedings of Machine Learning Research*, 5062–5071 (PMLR, 2019).

[24] Schmidhuber, J. Formal theory of creativity, fun, and intrinsic motivation (1990–2010). *IEEE Transactions on Autonomous Mental Development* **2**, 230–247 (2010).

[25] Sekar, R. *et al.* Planning to explore via self-supervised world models. In III, H. D. & Singh, A. (eds.) *Proceedings of the 37th International Conference on Machine Learning*, vol. 119 of *Proceedings of Machine Learning Research*, 8583–8592 (PMLR, 2020).

[26] Pathak, D., Agrawal, P., Efros, A. A. & Darrell, T. Curiosity-driven exploration by self-supervised prediction. In *Proceedings of the 34th International Conference on Machine Learning - Volume 70*, ICML'17, 2778–2787 (JMLR.org, 2017).

[27] Burda, Y. *et al.* Large-scale study of curiosity-driven learning. In *International Conference on Learning Representations* (2019).

[28] Hastie, T., Tibshirani, R., Friedman, J. H. & Friedman, J. H. *The elements of statistical learning: data mining, inference, and prediction*, vol. 2 (Springer, 2009).

[29] Rowan, T. H. *Functional stability analysis of numerical algorithms.* Ph.D. thesis, The University of Texas at Austin (1990).

[30] Nocedal, J. & Wright, S. J. *Numerical optimization* (Springer New York, NY, 2006).

[31] Piray, P., Dezfouli, A., Heskes, T., Frank, M. J. & Daw, N. D. Hierarchical bayesian inference for concurrent model fitting and comparison for group studies. *PLOS Computational Biology* **15**, e1007043 (2019).

[32] Rigoux, L., Stephan, K. E., Friston, K. J. & Daunizeau, J. Bayesian model selection for group studies—revisited. *NeuroImage* **84**, 971–985 (2014).

[33] Efron, B. & Hastie, T. *Computer age statistical inference* (Cambridge University Press, 2016).

[34] Giron, A. P. *et al.* Developmental changes in exploration resemble stochastic optimization. *Nature Human Behaviour* **7**, 1955–1967 (2023).

[35] Daw, N. Trial-by-trial data analysis using computational models. *Decision making, affect, and learning: Attention and performance XXIII* **23** (2011).

[36] Wilson, R. C. & Collins, A. G. Ten simple rules for the computational modeling of behavioral data. *eLife* **8**, e49547 (2019).

[37] McDougle, S. D. & Collins, A. G. Modeling the influence of working memory, reinforcement, and action uncertainty on reaction time and choice during instrumental learning. *Psychonomic Bulletin & Review* **28**, 20–39 (2021).

[38] Collins, A. G. & Frank, M. J. How much of reinforcement learning is working memory, not reinforcement learning? a behavioral, computational, and neurogenetic analysis. *European Journal of Neuroscience* **35**, 1024–1035 (2012).

[39] Gershman, S. J. & Blei, D. M. A tutorial on Bayesian nonparametric models. *Journal of Mathematical Psychology* **56**, 1–12 (2012).

[40] Teh, Y. W. *Dirichlet Process*, 280–287 (Springer US, Boston, MA, 2010).

[41] Blei, D. M. & Frazier, P. I. Distance dependent chinese restaurant processes. *Journal of Machine Learning Research* **12**, 2461–2488 (2011). URL http://jmlr.org/papers/v12/blei11a.html.

[42] Meyniel, F., Maheu, M. & Dehaene, S. Human inferences about sequences: A minimal transition probability model. *PLoS Computational Biology* **12**, 1–26 (2016).

[43] Puterman, M. L. *Markov decision processes: discrete stochastic dynamic programming* (John Wiley & Sons, 1994).

[44] Brea, J. Is prioritized sweeping the better episodic control? *arXiv preprint arXiv:1711.06677* (2017).

[45] Poli, F., O'Reilly, J. X., Mars, R. B. & Hunnius, S. Curiosity and the dynamics of optimal exploration. *Trends in Cognitive Sciences* **28**, 441–453 (2024).
